# Prediction of continuous and discrete kinetic parameters in horses from inertial measurement units data using recurrent artificial neural networks

**DOI:** 10.1038/s41598-023-27899-4

**Published:** 2023-01-13

**Authors:** J. I. M. Parmentier, S. Bosch, B. J. van der Zwaag, M. A. Weishaupt, A. I. Gmel, P. J. M. Havinga, P. R. van Weeren, F. M. Serra Braganca

**Affiliations:** 1 Department of Clinical Sciences, Faculty of Veterinary Medicine, Utrecht University, 3584 CM Utrecht, The Netherlands; 2 Inertia Technology B.V., 7521 AG Enschede, The Netherlands; 3 Pervasive Systems Group, Department of Computer Science, University of Twente, 7522 NB Enschede, The Netherlands; 4 Equine Department, Vetsuisse Faculty, University of Zürich, Winterhurerstrasse 260, Zurich, Switzerland; 5 Animal GenoPhenomics, Agroscope, 1725 Posieux, Switzerland

**Keywords:** Computer science, Biomechanics

## Abstract

Vertical ground reaction force (GRFz) measurements are the best tool for assessing horses' weight-bearing lameness. However, collection of these data is often impractical for clinical use. This study evaluates GRFz predicted using data from body-mounted IMUs and long short-term memory recurrent neural networks (LSTM-RNN). Twenty-four clinically sound horses, equipped with IMUs on the upper-body (UB) and each limb, walked and trotted on a GRFz measuring treadmill (TiF). Both systems were time-synchronised. Data from randomly selected 16, 4, and 4 horses formed training, validation, and test datasets, respectively. LSTM-RNN with different input sets (All, Limbs, UB, Sacrum, or Withers) were trained to predict GRFz curves or peak-GRFz. Our models could predict GRFz shapes at both gaits with RMSE below 0.40 N.kg^−1^. The best peak-GRFz values were obtained when extracted from the predicted curves by the all dataset. For both GRFz curves and peak-GRFz values, predictions made with the All or UB datasets were systematically better than with the Limbs dataset, showing the importance of including upper-body kinematic information for kinetic parameters predictions. More data should be gathered to confirm the usability of LSTM-RNN for GRFz predictions, as they highly depend on factors like speed, gait, and the presence of weight-bearing lameness.

## Introduction

Lameness in horses can be defined as an alteration of the normal gait due to a functional or structural disorder of the locomotor system and is often attributed to orthopaedic pain^1^. Therefore, quantitative gait analysis in horses is an essential process both in a clinical and research setting, as it is used as a tool in lameness diagnostic workup^2^. Kinetic analysis of the vertical ground reaction force (GRFz) is considered the 'gold standard' for quantifying weight-bearing lameness in horses^3^. Changes in GRFz curve shape and in peak GRFz (pGRFz) difference between limb pairs (loading asymmetry) can be used as an indicator of weight-bearing lameness in horses^4–8^ and other quadrupedal mammals^9–12^.

Equine GRFz is traditionally obtained with stationary force and pressure-plate systems^13–15^, or an instrumented treadmill (TiF, University of Zurich)^16^. These kinetic methods are well established and validated but remain sparsely used in the clinical setting, as they are cumbersome due to laborious data collection and processing requirements, especially the force and pressure-plate systems. TiF allows data collection of consecutive strides of all limbs simultaneously, but it requires acclimatisation and training to treadmill locomotion. Currently there is only one such instrument available worldwide, and it only records the vertical component of the GRF. Moreover, neither system can be used to retrieve data on different types of ground typically used for training or competitions. In an attempt to overcome these limitations, instrumented horseshoes have been developed by several research groups, which allow for more versatile measurements of GRFz. For example, they were used to collect three-dimensional (3D) GRF of trotters training on the sand beach^17^ or during jumping exercise^18^. The instrumented horseshoes overcome most of the stationary force plate and TiF systems' constraints, but they remain impractical, expensive, bulky and heavy, thus influencing the lower limb kinematics^19–21^ and being restricted to research settings only.

Several kinematic measurement methods such as optical motion capture (OMC), accelerometry or inertial measurement unit (IMU) technologies have gained popularity. They have proven to be practical, affordable and user-friendly methods for quantifying locomotion in horses^2,22^ both in research and clinical settings. Kinematic methods mainly focus on quantifying movement adaptations when horses experience pain. Most methods rely on quantifying movement asymmetry as an outcome variable for the degree of lameness. These asymmetries are caused by adaptation strategies to reduce the loading of the lame limb(s)^5,7^. Therefore, kinematics are only an indirect quantifier of weight-bearing lameness, while the measurement of the GRFz remains the gold standard, as it precisely measures the loading of each limb.

Given this situation, there is a clear need for indirect measurements and estimations of equine GRFz and pGRFz difference values that are more suitable for data collection outside the laboratory environment, such as the clinical setting. Witte et al.^23^ used hoof-mounted accelerometers to calculate the ratio between the stance and stride durations, called duty-factor (DF), and estimate individual limbs pGRFz at walk, trot and canter, based on an equation proposed by Alexander et al.^24^. Their method slightly overestimated pGRFz at the walk with a mean error of 0.8 N.kg^−1^, corresponding to 13% of pGRFz. In addition, they did not specify which peak (first or second) they extracted from the GRFz curves. They obtained good results at trot, with a mean error of − 0.3 N.kg^−1^ corresponding to 3% of pGRFz. Disadvantages of their method are that it is limited to the pGRFz estimation and relies on hoof-mounted accelerometers, which require highly resistant equipment due to the high impacts experienced on the distal limb during locomotion. Bobbert et al.^25^ developed a biomechanical model based on whole-body OMC kinematic data to predict the GRFz curves of individual limbs from horses walking and trotting on the instrumented treadmill. Their method was able to estimate the expected GRFz curve shape at trot. However, the GRFz curve shape was not well reproduced at walk, especially for the hindlimbs. More recently, Roepstorff et al.^26^ modelled front and hindlimb pGRFz differences using head, withers and sacrum kinematic variables derived from OMC data. They showed that accelerometry-based models resulted in a better fit and that asymmetry timing was related to pGRFz differences.

There are many studies on human subjects using machine learning to predict kinetic parameters^27^. For example, Alcantara et al.^28^ predicted continuous GRFz from accelerometers data in running tasks, using long-short term memory recurrent neural network (LSTM-RNN)^29^. However, equine locomotion differs from human locomotion because it involves single limb, bipedal or tripedal support phases at walk and single limb or bipedal support phases at trot, meaning that the loads are distributed over one, two, or three limbs for each moment of the gait. Unipedal support phases can be found only for a short duration at trot^30^, or at canter and gallop^31,32^ and in some additional gaits in a number of specific horse breeds, like the tölt in the Icelandic horse^33–35^. This characteristic highly increases the limb loading patterns possibilities, and the individual possible compensation patterns make the prediction of GRFz from kinematics a highly complex problem.

While machine learning and deep learning techniques have been applied to other equine applications such as gait^36^ and activity recognition^37^, only one study showed that equine ground reaction forces could be predicted. Savelberg et al.^38^ successfully trained an artificial neural network (ANN) to predict 3D GRF profiles based on hoof wall 3D displacements measured with strain gauges. While innovative, this method remained impractical as it required precise gluing of the strain gauges and a force plate as a reference method and was applied to one horse only. More recently, Mouloodi et al.^39^ successfully predicted mechanical hoof strains from a hoof-mounted IMU in a galloping racehorse, using a horseshoe instrumented with a strain gauge as reference data and feedforward time-series (dynamic) artificial neural networks. Their work showed promising results with R-values exceeding 0.98 with their best models, although applied to a single horse. Deep learning models have also been used to detect and quantify lameness, with different kinematic input types (e.g., upper-body and limbs displacements) and sensor types (video or IMU), applied to existing unilateral lameness^40,41^ or induced gait abnormalities^42^. These works show the potential application of deep learning models to detect and understand equine locomotion.

This study aims to evaluate the ability of LSTM-RNN to:

1. Accurately predict continuous equine GRFz based on whole-body IMU signals and LSTM-RNN during treadmill locomotion;
2. Accurately predict the discrete GRFz parameters (i.e., pGRFz and time-to-pGRFz) with LSTM-RNN and compare them to extracted parameters from the LSTM-RNN predicted continuous GRFz;
3. Evaluate left–right pGRFz symmetry indices (SI) for both front (SI-Front) and hind (SI-Hind) limb pairs, calculated for both the extracted and predicted pGRFz;
4. Investigate for all the above parameters the optimal IMU nodes set required to achieve the best predictions.

We hypothesised that accurate GRFz curve shapes could be obtained for both bipedal and tripedal support with LSTM-RNN, with better results obtained when all nodes are used for training the models.

## Results

### Continuous GRFz

**Figure 1:**
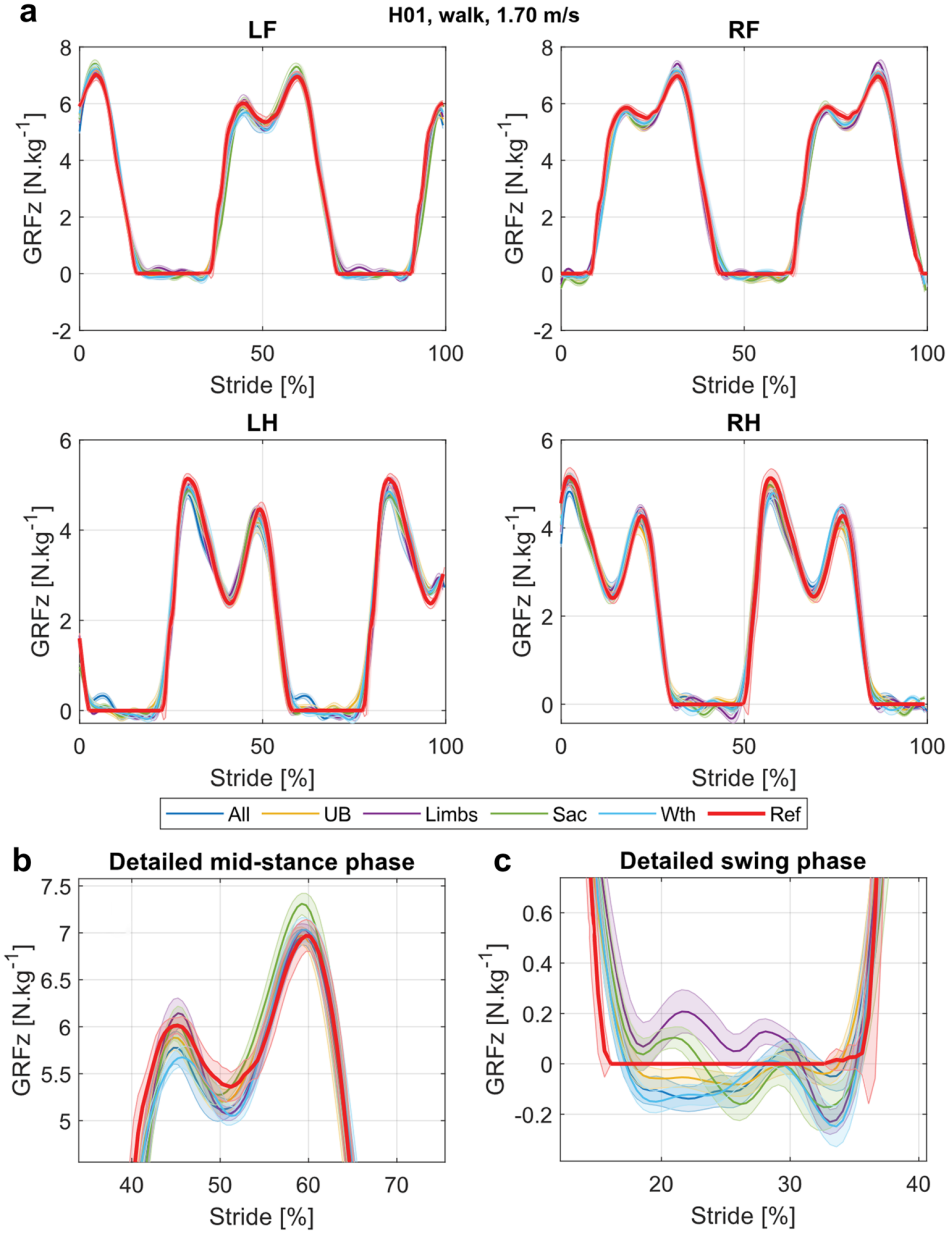
Example of GRFz curves predicted by the different nodes sets for the walk data, at 1.70 m/s. (**a**) GRFz for all limbs. The average strides are displayed as a bold line and standard deviations are shown by thin lines and shaded areas. The reference curves are shown in red (Ref: TiF reference), the curves predicted by models trained with all nodes (All: head, withers, sacrum and limb nodes) in orange, with upper-body nodes (UB: head, withers and sacrum nodes) in yellow, with limb nodes (Limbs: limb nodes) in purple, with the sacrum node (Sac: sacrum node) in green and with the withers node (Wth: withers node) in light blue. *LF* left front, *RF* right front, *LH* left hind, *RH* right hind. (**b**) Detailed comparison of the predictions during the stance phase of the LF limb. (**c**) Detailed comparison of the predictions during the swing phase of the LF limb.

**Figure 2:**
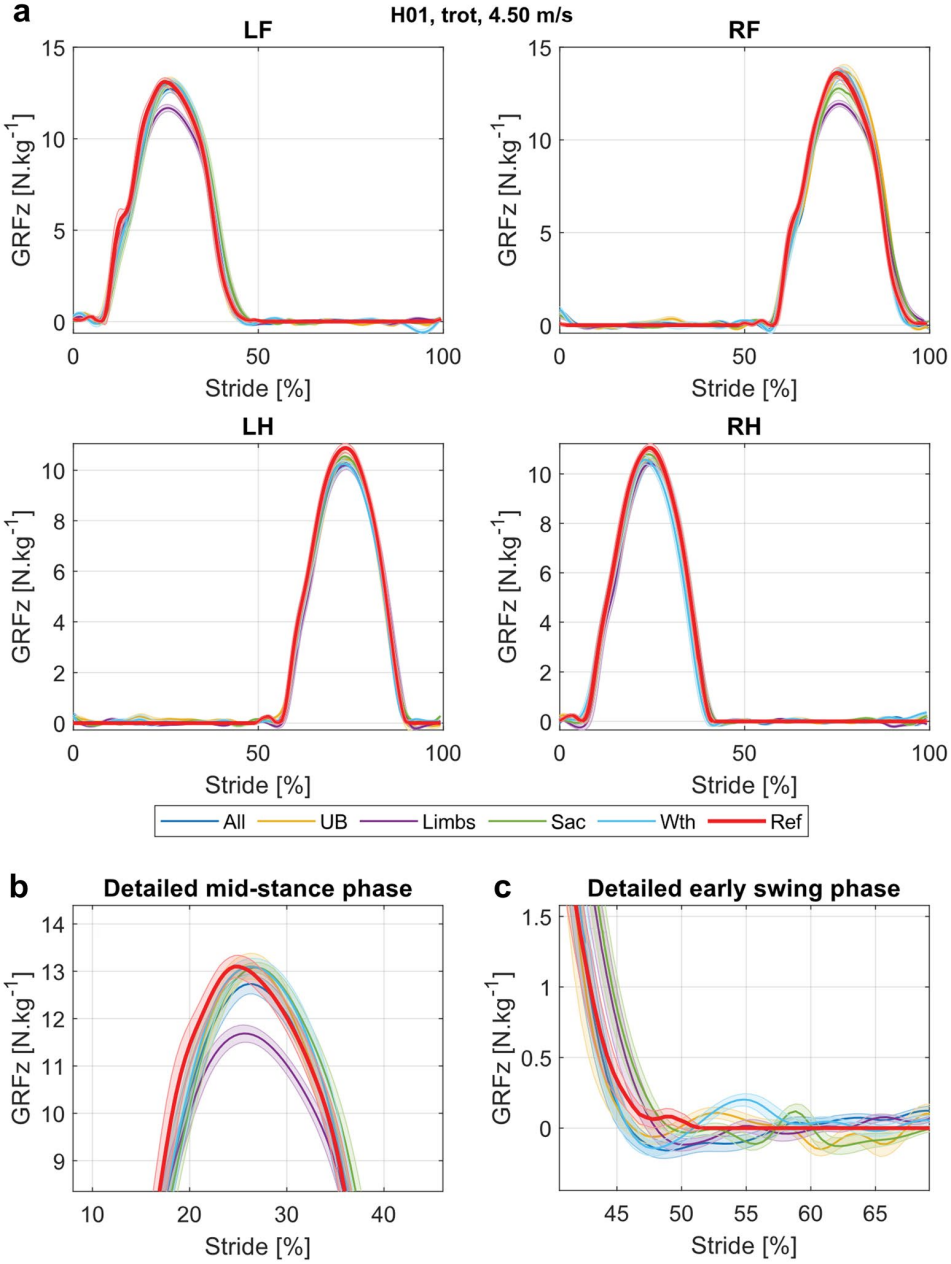
Example of GRFz curves predicted by the nodes sets for the trot data, at 4.50 m/s. (**a**) GRFz for all limbs. The average strides are displayed as a bold line and standard deviations are shown by thin lines and shaded areas. The reference curves are shown in red (Ref: TiF reference), the curves predicted by models trained with all nodes (All: head, withers, sacrum and limb nodes) in orange, with upper-body nodes (UB: head, withers and sacrum nodes) in yellow, with limb nodes (Limbs: limb nodes) in purple, with the sacrum node (Sac: sacrum node) in green and with the withers node (Wth: withers node) in light blue. *LF* left front, *RF* right front, *LH* left hind, *RH* right hind. (**b**) Detailed comparison of the predictions during the stance phase of the LF limb. (**c**) Detailed comparison of the predictions during the swing phase of the LF limb.

Upon visual assessment of the models' output, all IMU nodes combinations were able to predict the characteristic GRFz curve shapes, namely the double-peak at walk, with a higher second peak for the front limbs and a higher first peak for the hind limbs and the single peak at trot (Figs. 1, 2). The footfall patterns were also correctly predicted, LH-LF-RH-RF at walk and (LH-RF)-(RH-LF) at trot. These findings are also supported by the extracted time-to-pGRFz (t-pGRFz), as shown in the Supplementary Materials (Supplementary Figs. S5 and S6). For both gaits, but especially at walk, the predicted GRFz were not null during the swing phase (Fig. 1c).

### Discrete pGRFz

**Figure 3:**
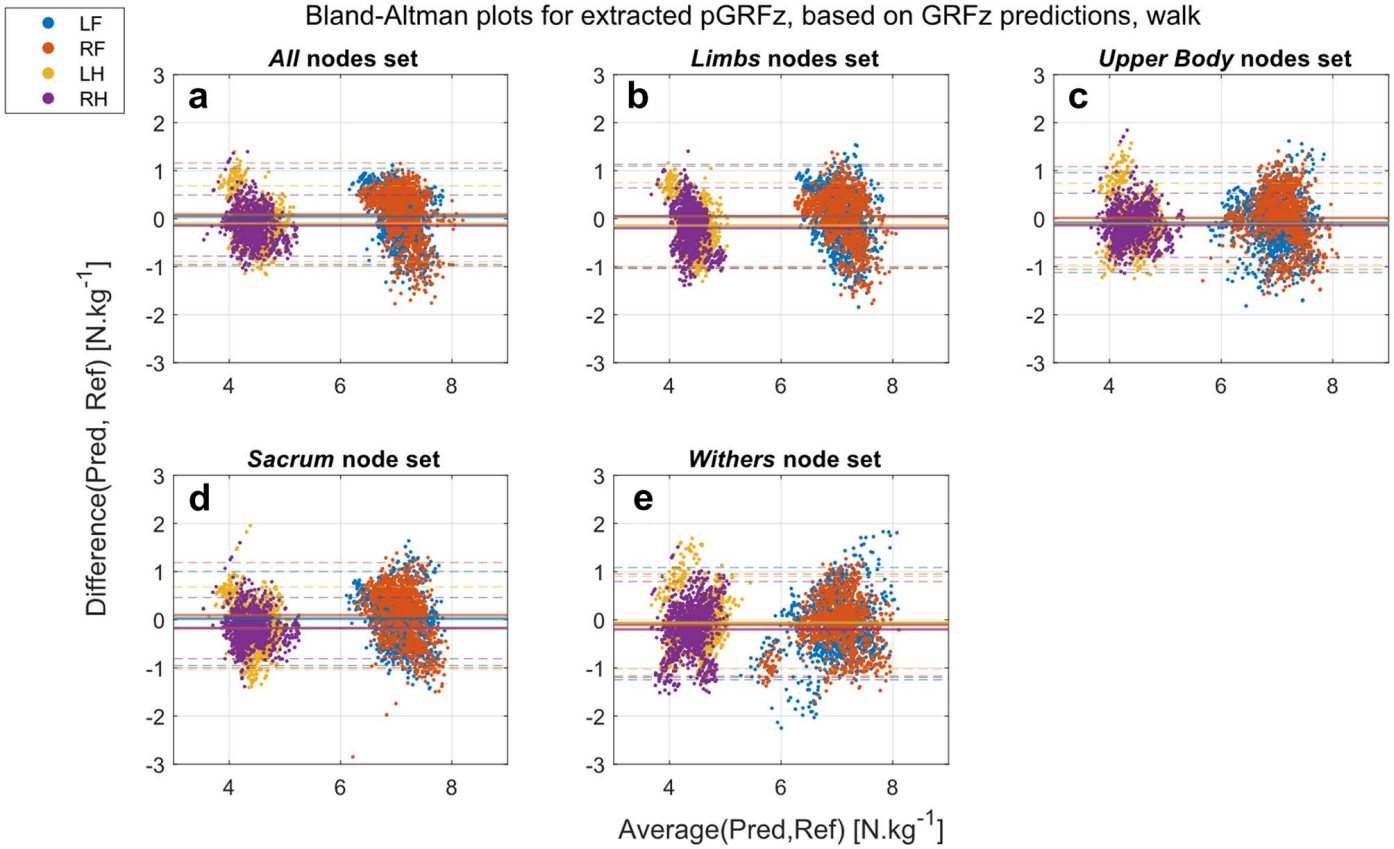
Bland–Altman plots of pGRFz extracted from the GRFz curves predicted by the different nodes sets at walk (Pred) against the reference values extracted from the TiF data (Ref): (**a**) All (head, withers, sacrum, and limb nodes); (**b**) Limbs (limb nodes); (**c**) Upper-body (head, withers and sacrum nodes); (**d**) Sacrum (sacrum node); (**e**) Withers (withers node). *LF* left front, *RF* right front, *LH* left hind, *RH* right hind.

**Figure 4:**
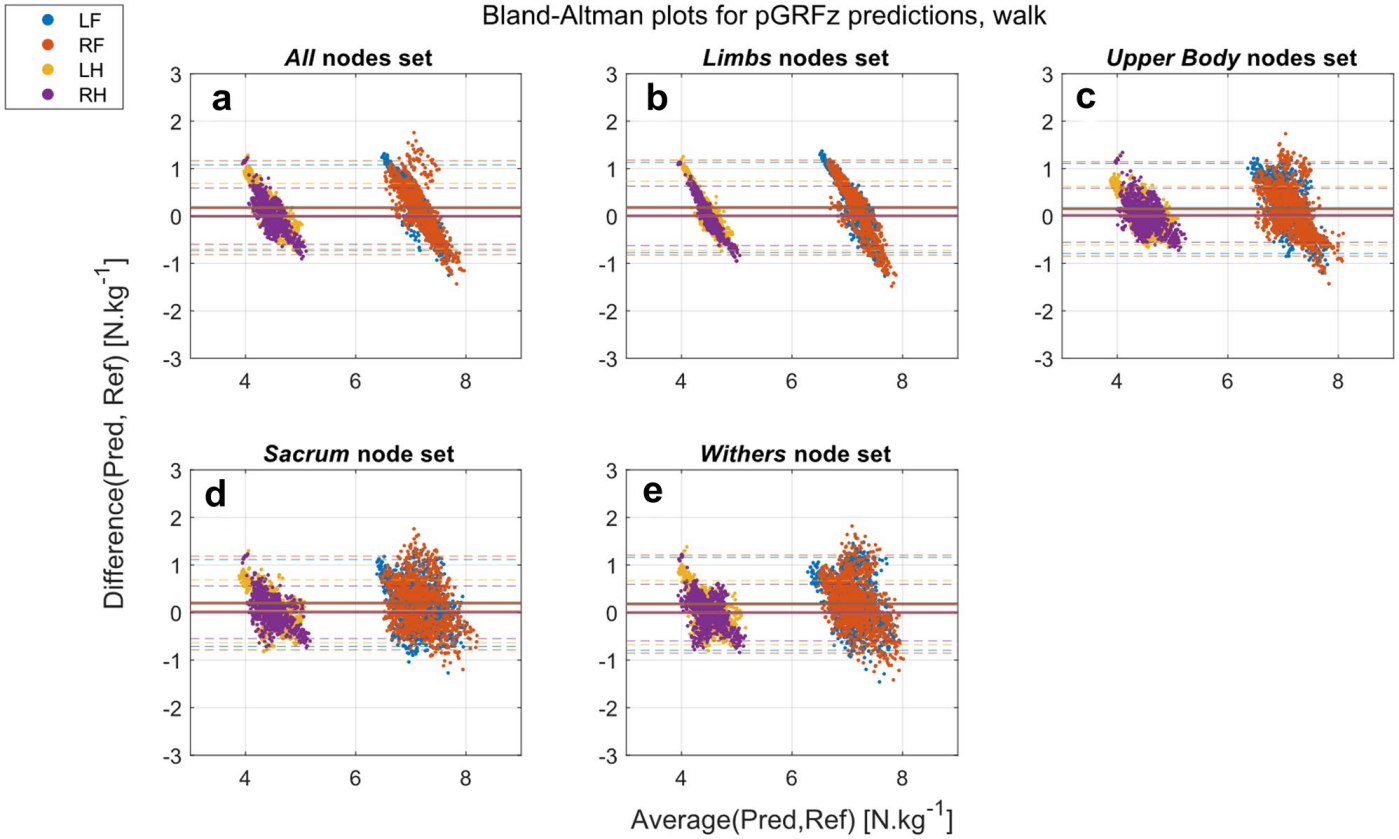
Bland–Altman plots of the predicted pGRFz by the different nodes sets at walk (Pred) against the reference values extracted from the TiF data (Ref): (**a**) All (head, withers, sacrum, and limb nodes); (**b**) Limbs (limb nodes); (**c**) Upper-body (head, withers and sacrum nodes); (**d**) Sacrum (sacrum node); (**e**) Withers (withers node). *LF* left front, *RF* right front, *LH* left hind, *RH* right hind.

For the walk dataset, the extracted and predicted pGRFz have achieved good results (biases close to 0), showing higher accuracies, regardless of the limb and the nodes set used (Figs. 3, 4). The predicted pGRFz presents a points cloud following a line with a negative trend (Fig. 4a:d). This shows the low ability of this method to correctly predict the pGRFz at walk, regardless of the input, as shown in the correlation graphs in the Supplementary Materials, Fig. S2. pGRFz extracted from the predicted GRFz curves (Fig. 3) show better Bland–Altman patterns and thus better prediction results, which is also supported by the correlation graphs in the Supplementary Materials (Supplementary Fig. S1).

**Figure 5:**
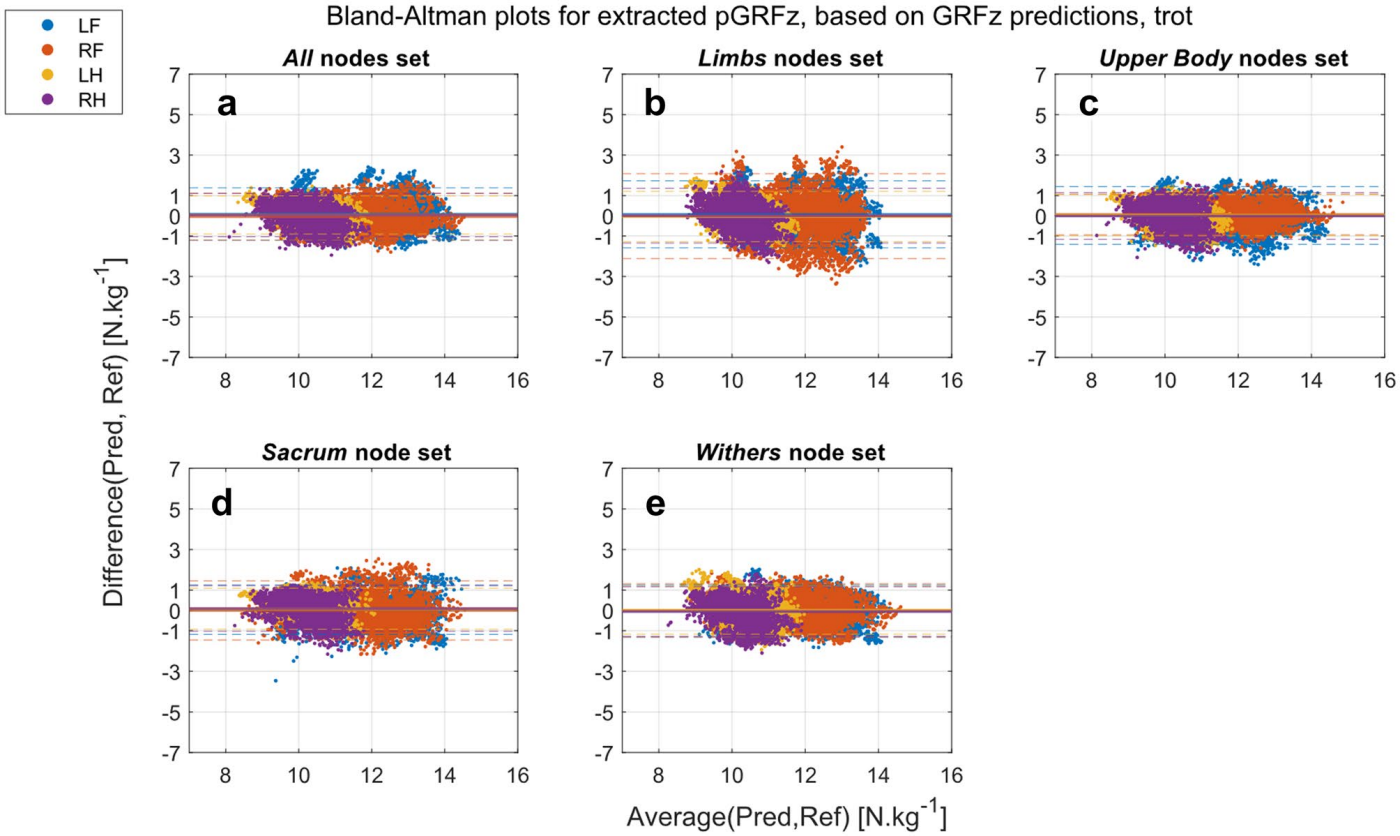
Bland–Altman plots of pGRFz extracted from the GRFz curves predicted by the different nodes sets at trot (Pred) against the reference values extracted from the TiF data (Ref): (**a**) All (head, withers, sacrum, and limb nodes); (**b**) Limbs (limb nodes); (**c**) Upper-body (head, withers and sacrum nodes); (**d**) Sacrum (sacrum node); (**e**) Withers (withers node). *LF* left front, *RF* right front, *LH* left hind, *RH* right hind.

**Figure 6:**
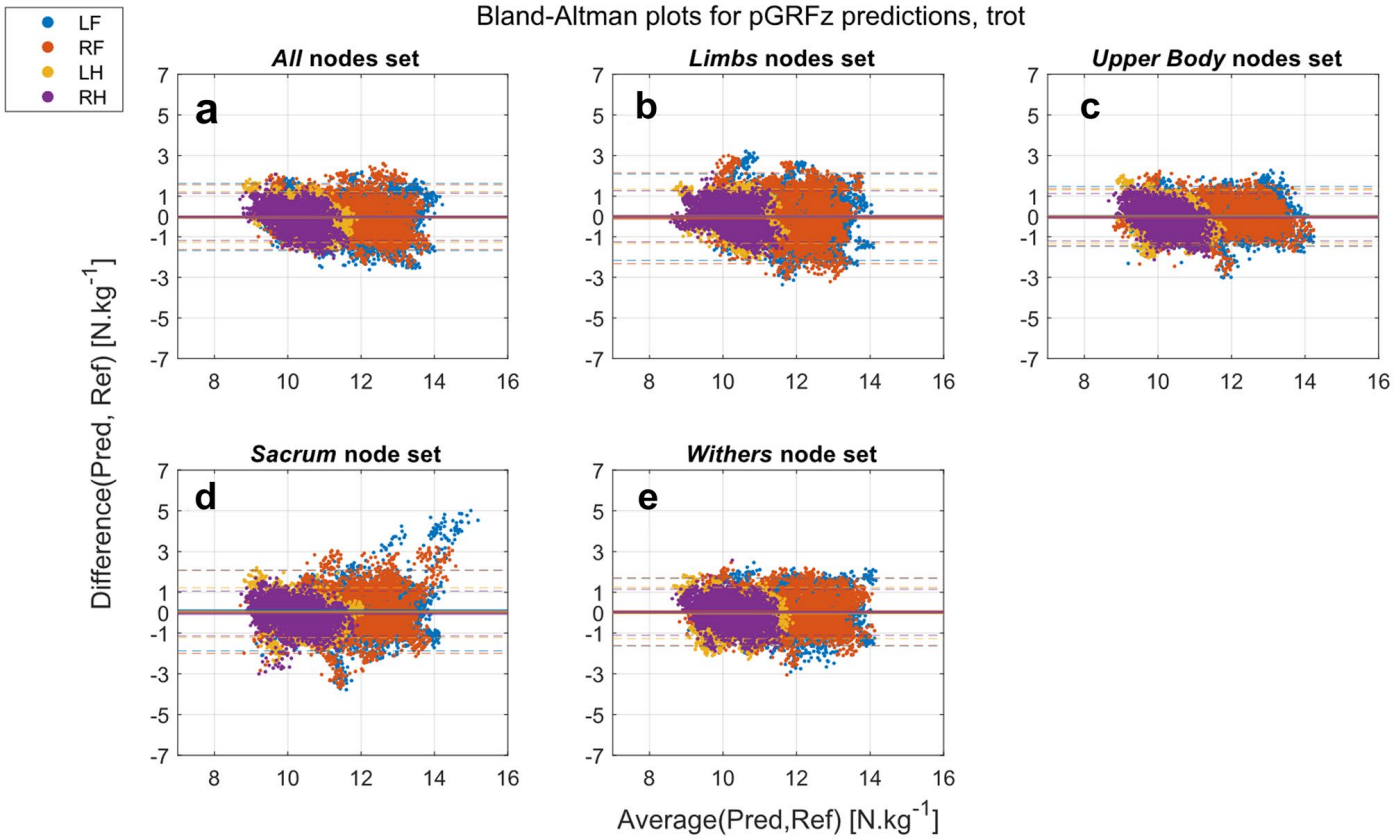
Bland–Altman plots of the predicted pGRFz by different nodes sets at trot (Pred) against the reference values extracted from the TiF data (Ref): (**a**) All (head, withers, sacrum, and limbs nodes); (**b**) Limbs (limb nodes); (**c**) Upper-body (head, withers and sacrum nodes); (**d**) Sacrum (sacrum node); (**e**) Withers (withers node). *LF* left front, *RF* right front, *LH* left hind, *RH* right hind.

For the trot dataset, the biases were also low for all extracted and predicted pGRFz and, regardless of the nodes set used (Figs. 5, 6, Supplementary Table 1). Limits of agreements were wider for the predicted pGRFz than the extracted pGRFz (Supplementary Table 1).

### pGRFz symmetry indices

For the walk dataset, symmetry indices (SI) were better estimated when calculated with the extracted pGRFz (Supplementary Material, Fig. S7), shown by lower biases and narrower upper- and lower-bounds, whereas the SI estimated with the predicted pGRFz also displayed the same negative trend (Supplementary Material, Fig. S8) seen in the pGRFz predictions (Fig. 4). Extracted SI-Front and -Hind have relatively low biases but wide upper- and lower-bounds (Supplementary Table 2).

At trot, the best SI-Front and -Hind were obtained when calculated from the GRFz curves predicted with the All nodes set (Supplementary Material, Fig. S9a), and presented an overall better correlation with the reference SI (Supplementary Material, Fig. S13). The limits of agreement are also better but rather large (Supplementary Table 2). The SI based on pGRFz predictions also presented a negative trend (Supplementary Material, Fig. S10).

### Nodes sets comparison

#### GRFz curves

**Figure 7:**
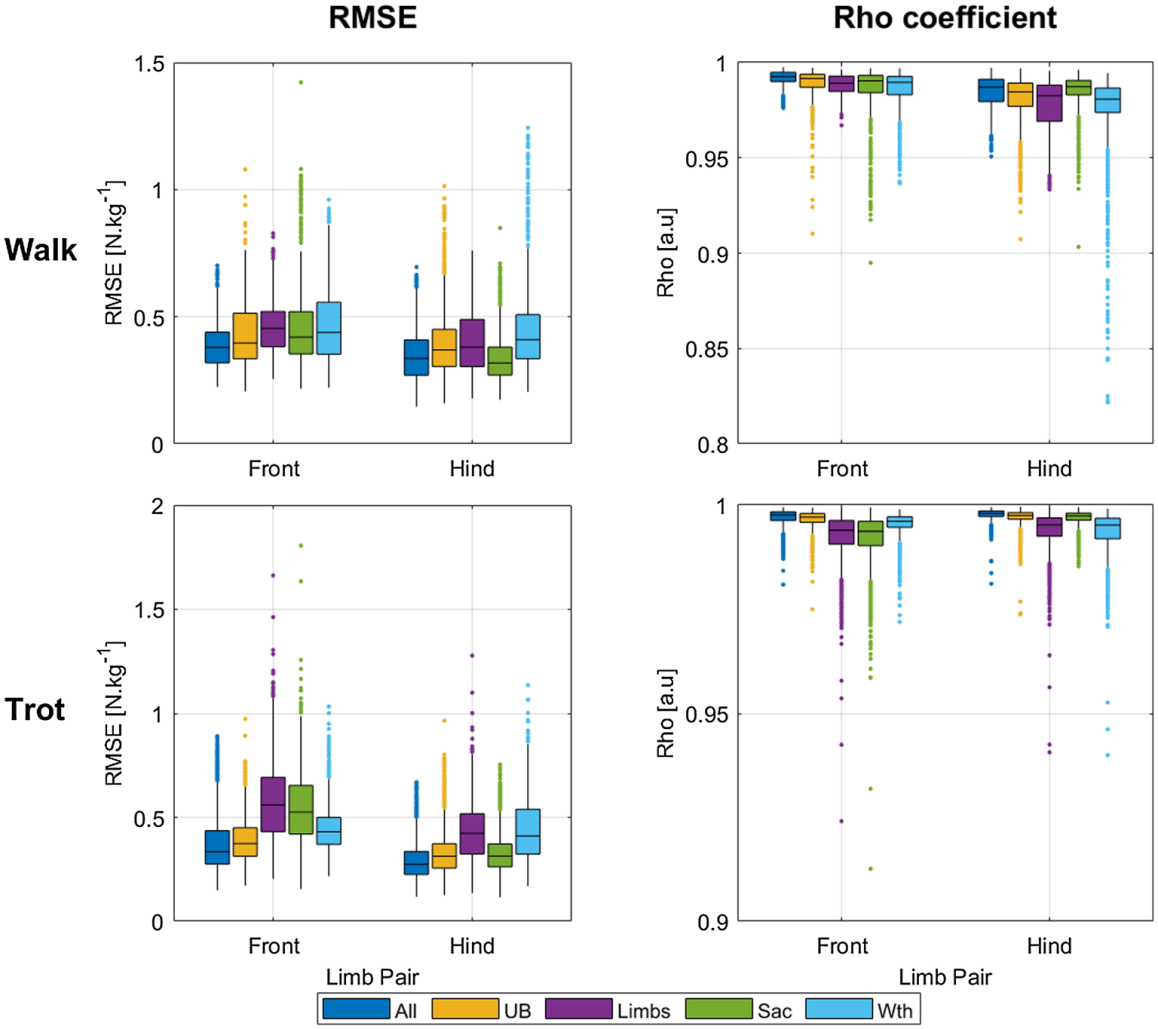
Boxplots of the RMSE (left column) and Rho correlation coefficients (right column) calculated for the GRFz curves predictions at walk (upper row) and trot (lower row), predicted by models trained with all nodes (All: head, withers, sacum and limb nodes) in dark blue, with upper-body nodes (UB: head, withers and sacrum nodes) in yellow, with limb nodes (Limbs: limb nodes) in purple, with the sacrum node (Sac: sacrum node) in green, and with the withers node (Wth: withers node) in light blue.

Overall, the best predictions were obtained with the All nodes set for both gaits and limb pairs, with median RMSE values under 0.40 and median rho correlation coefficients above 0.99. For both gaits, the predictions were better when using the UB set than the Limbs set. When using only one node at trot, front limb predictions were better with the Withers (Wth) set, whereas the hind limb predictions were better with the Sacrum (Sac) set. At walk, hind limb GRFz predictions were better when using the Sac set whereas the Wth set did not perform better than the Limbs set for the front limb GRFz (Fig. 7).

#### pGRFz values

Overall, the best extracted pGRFz results (smallest biases and smallest limits of agreement ranges) were obtained with the UB and All nodes sets, for both gaits and both limb pairs. At walk, the worst results were obtained with the Wth and the Limbs nodes sets (Supplementary Table 1), while at trot the Limbs nodes set presented the widest limits of agreement ([3.79, 2.60] N.kg^−1^ for the front and hind limbs, respectively).

At walk, the pGRFz values had smaller limits of agreement when using the All and Sac nodes set for the front limbs and the UB or Sac nodes sets for the hind limbs (Supplementary Table 1). The biases were smaller for the hind limbs than for the front limbs with all the nodes sets used. At trot, the smallest bias and narrowest limits of agreement for the front limbs were obtained with the UB nodes set (− 0.01 [− 1.45; 1.43] N.kg^−1^) where the Limbs nodes set had the widest limits of agreement (4.37 N.kg^−1^). The Sac node set presented the best pGRFz predictions for the hind limbs, with a range of agreement of 2.33 N.kg^−1^, whereas the Limbs set had a range of 2.59 N.kg^−1^.

## Discussion

In this study, we demonstrated that we could accurately (low RMSE and high rho coefficient values) predict continuous GRFz from raw IMU data using a machine learning approach in horses at walk and trot. Furthermore, our proposed models can be used to overcome some of the limitations (portability, useability) of using force-plates to measure GRFz, and we were able to improve the accuracy of these predictions based on some of the previously published methods.

### Continuous GRFz

Our first goal was to obtain GRFz curves based on the IMU signals. All LSTM-RNN models were able to predict the correct GRFz curve shapes at both walk and trot, regardless of the nodes set used. To the best of our knowledge, this is the first time that equine GRFz curves and associated parameters are predicted using RNNs. Savelberg et al. predicted continuous GRFz based on hoof wall deformation using an ANN at walk and trot. Their dataset was constructed for one subject only and showed promising results for the walk GRFz estimations (cross-correlation coefficient: 0.98), but poorer results were obtained for the trot GRFz estimations (cross-correlation coefficient: 0.60). Our results showed promising GRFz predictions in sound horses with rho correlation coefficients above 0.90 for all gaits and limbs, and median RMSE below 0.5 N.kg^−1^ when the all dataset was used.

Similar works in the human literature showed RMSE of 0.27 N.kg^−1^ when training and testing for single subject^43^. When training for multiple subjects, their methods showed an increase in RMSE and a decrease in rho correlation coefficients, indicating poorer results. Our LSTM-RNN were mainly able to predict the recognisable GRFz shape at walk, which in most strides, both front and hind limbs comprise two peaks and one dip^14,44,45^. Kinematic studies showed that the dip occurs during tripedal support at walk while the peaks occur during bipedal support^46,47^. Weishaupt et al.^48^ showed that the presence of the dip is also dependent on the walking speed, and it tends to disappear with lower speeds. We tried to incorporate this speed dependency by adding data measured at different speeds in our training and testing datasets for both gaits. Examples of predicted GRFz at different speeds are shown in Supplementary Materials, Figs. S15 and S16.

### Discrete pGRFz and SI

Our second goal was to evaluate the direct prediction of peak GRFz values by LSTM-RNN compared to extracting these values indirectly from the continuous predicted GRFz signals. Front limb lameness induction models have shown that for walk, pGRFz values decrease for the lame limb and increase both in the contralateral limb and in the diagonal hindlimb^49,50^. At trot, pGRFz decreased in the lame front or hind limb without significantly overloading the contralateral limb^5,7^. In moderate forelimb lameness, studies have shown a redistribution of the weight towards the rear with an increase loading of the diagonal hindlimb^5,6^. pGRFz are thus an essential parameter to obtain from kinetic analysis, especially in the case of lameness quantification.

At walk, the best pGRFz prediction method was to extract the pGRFz from the LSTM-RNN predicted curves. The Bland–Altman analysis showed that the directly predicted pGRFz did not have good accuracy and tended to provide values within a limited range. For the pGRFz predicted by the LSTM-RNN, it is possible that the used stride split method confused the network, preventing it from identifying the correct peak among the two-peak bells and the different available stance phases. This hypothesis is supported by the fact that better results were obtained when the pGRFz were extracted from the predicted GRFz curves. Since walk contains bipedal and tripedal support phases, it is more difficult for the network to understand which limb contributes to the pGRFz. Training for each limb individually instead of training for the four limbs could positively impact our method's performance. Further experiments are needed to test these hypotheses.

At trot, all prediction methods showed better accuracies than at walk. The pGRFz extracted from the predicted curves had more promising results than the pGRFz predicted by LSTM-RNN, with lower biases and ranges. This might show the better potential of LSTM-RNN to predict continuous variables rather than discrete output variables.

Because of the potential application of our method for lameness evaluation, we calculated the pGRFz SI indices for both pairs of limbs and evaluated them against the reference TiF data. The SI based on the LSTM-RNN predicted pGRFz had a low agreement with the ones calculated with the TiF data, for both gaits. The SI calculated with the pGRFz extracted from the LSTM-RNN predicted GRFz showed more promising results, especially at the trot for the all nodes set.

Since our dataset included only sound horses for which our method seemed to overestimate SI, our models have had no comparisons between sound and lame horses (hence low and high SI values) to learn from. We thus speculate that training LSTM-RNN to recognise and estimate asymmetries in horses with weight-bearing lameness could improve the accuracy of SI values predictions. However, our models would not be directly applicable to lameness quantification in their current state.

### Nodes sets comparison

The final aim of this study was to define which nodes were crucial for the prediction of the GRFz curves and parameters. Our results showed that regardless of the predicted modality, the best results were always obtained when the upper-body nodes were used, either with the All nodes set or just the UB one. Based on the DF method hypotheses from Witte et al.^23^, we expected that the Limbs nodes set would have predicted the pGRFz accurately, but accuracies obtained with nodes sets including one upper-body nodes only (i.e., Sac or Wth) were better. This correlates with the results from Bobbert et al.^25^, who calculated individual limbs GRFz based on whole-body kinematics and the acceleration of the centre of mass (COM). Hobbs and Clayton^51^ reported that GRFz controls the vertical movement of the COM, for which the upper-body accelerations and rotation rates used in our study could be an approximation. Roepstorff et al.^26^ showed that upper-body acceleration asymmetry variables could be used to model differences in pGRFz, and that timing of these asymmetries is also important. Therefore, we can hypothesise that if the kinematics of the upper-body are known, the LSTM-RNN can trace their causality back to kinetic parameters and, in the future, model pGRFz differences that can be used for (early) weight-bearing lameness detection.

### Limitations

Our study is mainly limited by the low biological variability between the horses used. All were from the Franches–Montagnes breed, with similar height and weight, which influences the GRFz ranges of values and motion patterns. Moreover, the signals were measured during treadmill locomotion, which slightly differs from overground locomotion. Especially, Buchner et al.^52^ have shown that treadmill locomotion increased the relative stance duration and decreased the relative swing phase duration, influencing the load redistribution pattern. Thus, our models and results cannot be directly transferred to other breeds or to overground and ridden locomotion yet. Furthermore, the models were developed with healthy and sound horse data. Weight-bearing lameness will invariably change the motion patterns and thus load distribution over the limbs^3,5–7^. Nevertheless, the models should be extendable to the other cited conditions provided that proper data collection is conducted. Lastly, our work focused on GRFz only due to the limitations of the reference system used, the TiF. Other techniques, such as the instrumented force shoes, would enable the concurrent collection of vertical, anteroposterior and mediolateral GRF.

## Conclusion

Our results hence show that it is possible for machine learning to put kinematics and kinetics in relation to each other. This will be more than an essential asset for equine orthopaedics to understand the complexity of the biomechanical movement chains in horses. As alluded to earlier, kinetics are seen as the gold standard for the definition of weight-bearing lameness, which is by far the most common type of lameness seen in horses^2^. The problem for practical equine gait analysis and lameness detection is that no easily clinically applicable tools exist that can measure kinetics; the existing tools (force plate, instrumented force shoes, instrumented treadmill) are either too cumbersome, rarely available (force plates, instrumented force shoes, instrumented treadmill), or interfering themselves with the locomotion (instrumented force shoes, treadmill). The approach developed in this study allows for the accurate estimation of kinetics from upper-body and/or limb kinematics, which both can be measured with widely available tools that are already at a sharply increasing rate used in everyday clinical practice. Our results even show that models trained with nodes mounted on the upper-body (head, withers and sacrum) achieve higher accuracies predicting GRFz when compared to models using limb nodes. This finding supports using kinematic methods in a clinical setting using upper-body mounted nodes as these can reflect weight-bearing asymmetries in a more practical and daily usable solution when compared to kinetic methods and show the relative smaller contribution of limb kinematics to GRFz. This gives the method, therefore, the potential to significantly improve the quality and relevance of the outcome of quantitative (equine) gait analysis, making it a more than a valuable asset in equine orthopaedics, which is one of the most important disciplines in equine healthcare.

In conclusion, with our models, we could obtain GRFz curves based on IMU data, which offers new possibilities for locomotion analysis and, more specifically, lameness exams. Changes in GRFz data patterns are an essential indicator of weight-bearing lameness, and comparing changes in continuous GRFz curves to changes in discrete pGRFz and t-pGRFz would also be an interesting future study, as continuous data analysis provides more global information^53^. We also showed with our nodes set comparisons that there is an interconnection between kinetics and kinematics. Our results suggest that upper-body movements have a greater influence than limb movements on the GRFz, which needs to be considered during equine locomotion analyses. Even though force plates and instrumented treadmills remain the most accurate GRFz measurement tools, they are restricted to the laboratory environment and have their own technical limitations^2^. Our models enable accessing kinetic data in a more user-friendly manner, using field-applicable systems which will be a great asset for equine orthopaedics.

## Methods

### Subjects and ethics statement

Twenty-four Franches–Montagnes stallions (age 9 ± 4 years, body mass 526 ± 32 kg, height 156 ± 3 cm) that were clinically sound were used for this study. The Animal Health and Welfare Commission of the canton of Vaud (Switzerland) approved the experimental protocol prior to data collection (permission number VD3164), in accordance with the approved guidelines and protocols. Informed consent was obtained from the owner of the animals, and no human participants were included in this study.

### Materials

The experiment included three measurement systems: inertial measurement units containing 3D low-g and high-g accelerometers as well as a 3D gyroscope (ProMove-mini, Inertia Technology B.V., The Netherlands), high-speed instrumented (TiF, University of Zurich, Switzerland) treadmill (Mustang 2200, Ansorix Systems AG, Switzerland) and infrared optical motion capture (OMC) cameras system (Oqus 7+, Qualisys AB, Sweden) with skin mounted spherical reflective markers.

The IMU sampling rate was set at 200 Hz. The upper-body nodes (head, withers, sacrum) were configured with a range of ± 8 g and ± 100 g for the low-g and high-g 3D accelerometers, respectively. The limb accelerometers were configured with a range of ± 16 g and ± 200 g. The 3D gyroscope was configured with a range of ± 2000 dps for all nodes. The OMC system recorded at 200 Hz. The TiF allowed for tracing of the GRFz of individual limbs and was sampled at 512 Hz^16^.

### Experimental protocol and data acquisition

**Figure 8:**
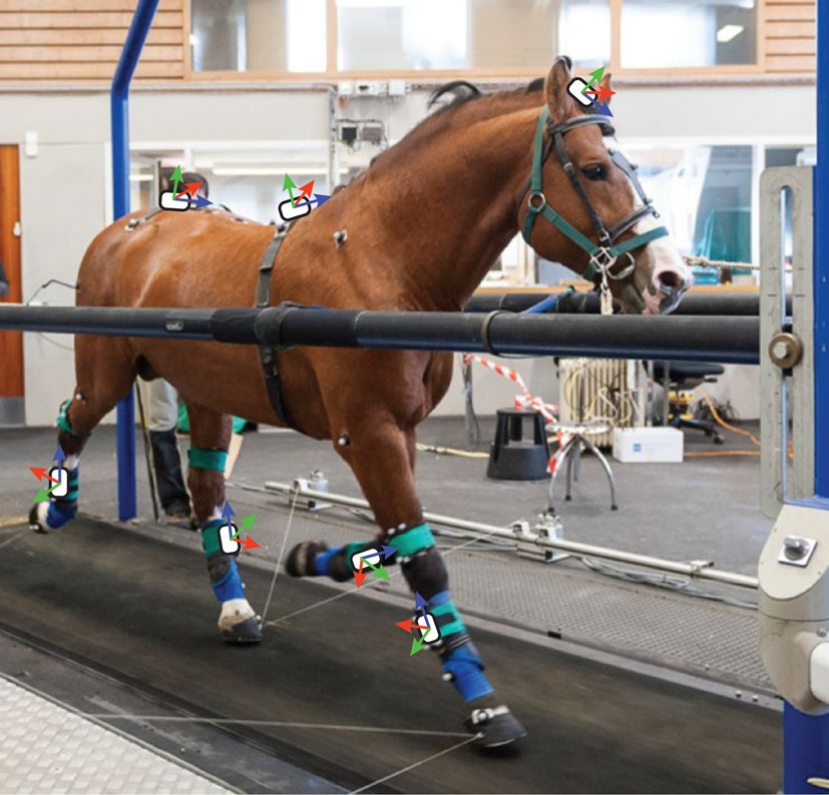
Horse on the TiF, equipped with reflective markers of the OMC system and the IMU nodes (white rectangles). Orientations of the nodes are shown with blue (x-axis), red (y-axis) and green (z-axis) arrows. Copyright: Erns A. Kehrli–Vetcom UZH.

The horses were habituated to the treadmill at the walk and trot, with eight to fourteen training sessions over a time span of six weeks prior to the measurements. Each subject was equipped with seven IMU nodes attached to the head, withers, sacrum, and lower limbs, as shown in Supplementary Materials, Fig. S13. The head node was fixed to a custom-made pouch sown on the bridle, the withers node was mounted on a girth, and the sacrum node was attached with double-sided tape over the tuber sacrale. The limb nodes were fixed by custom-made holsters to the lateral aspect of the metacarpus/metatarsus bones (Fig. 8).

Before and after the measurements, the horses stood still for IMU calibration, which is further described below. The horses were measured at two speeds at walk (1.7 m/s, 1.8 m/s) and four speeds at trot (3.3 m/s, 4.0 m/s, 4.5 m/s, 5.0 m/s), in incremental order. Data collection started once the horse's gait was stabilised and lasted for 20 s, ensuring the recording of at least fifteen consecutive strides.

### Data preparation

Data processing and analysis, and models training were performed in MATLAB 2021a (MathWorks, Natick, Massachusetts, USA).

### IMU signal processing

Raw IMU data was downloaded from the internal storage of IMU devices after the experiments, thereby avoiding any data loss due to wireless transmission.

The low-g accelerometers can present saturation at hoof impact, especially for the nodes located on the limbs. In order to keep the high acceleration information without decreasing the signal-to-noise ratio, a combined acceleration signal was computed from the low-g and high-g acceleration signals; we used the high-g data only around the areas of saturation with a threshold at 8 g, which was computed as:

$${\mathrm{acceleration}}_{\mathrm{merged}}=\left\{\begin{array}{l}{\mathrm{acceleration}}_{\mathrm{low}-\mathrm{g}} \; if \; acceleration<8g\\ {\mathrm{acceleration}}_{\mathrm{high}-\mathrm{g}} \; otherwise.\end{array}\right.$$

The gyroscope bias was compensated by determining that bias at the beginning of the experiment when the horse stood still for 10 s. The accelerometer was calibrated for scale using the gravity signal measured during that same period.

The calibrated signals were then used to construct the inputs for the LSTM-RNN.

### TiF signal processing and normalisation

TiF signal pre-processing was done in HP2 software (University of Zurich, Switzerland), allowing the obtention of the GRFz curves. Because the TiF was hardware-synchronised to the OMC system, time-synchronising the TiF GRFz signals to the IMU signals could be achieved indirectly by first synchronising the OMC system to the IMU system. The OMC to IMU synchronisation was performed by means of the synchronisation algorithm by Bosch et al.^54^, which uses the cross-correlation between angular velocity magnitude signals from the IMU and OMC systems. The synchronisation of the TiF GRFz signals was subsequently performed by resampling it using the parameters resulting from the OMC to IMU synchronisation. No other filtering or processing was performed on the TiF GRFz signals.

The TiF GRFz signals were then divided by the horse body mass and expressed in Newton per kilogram (N.kg^−1^).

### Data segmentation

The hoof event detection (hoof-on and hoof-off) was solely based on the IMU signals, using the previously described method^55^, as they would be the only ones available in situ. Data were segmented to have one complete stance phase and one full swing phase for each limb. Segmentation for each gait is described and illustrated below:

### Walk

The walk is a four-beat symmetrical gait, during which the horse will always have at least two hooves on the ground. The hoof impact sequence is left hind–left front–right hind–right front. Thus, we decided to define a window of walk data as the signals from left hind hoof-off to the next right front hoof-off, as seen in Supplementary Fig. S17a. This allows having one full stance phase and one complete swing phase per stride for each limb. The stance phases of interest in the defined stride are highlighted in red in the figure.

### Trot

The trot is a two-beat symmetrical gait, during which the horse has either none or the two diagonally opposed hooves in contact with the ground. Trot data windows were defined as the signals starting from the left hind hoof-off to the next left hind hoof-off, as seen in Supplementary Fig. S17b. The stance phases of interest in the defined stride are highlighted in red.

### Dataset construction

For each gait, a dataset was constructed following the steps described below.

The dataset construction is based on the window segmentation. For each horse and speed, the 20 s trial was split into all strides available in that segment. Then, the 3D merged acceleration signals and the 3D rotation rate signals of each node were normalised over 200 samples and vertically concatenated to form an input matrix of size (6 × N nodes) × 200 samples, depending on which nodes sets was used. Thus, each stride was represented by a matrix of (6 × N nodes) features of length 200, as shown in Supplementary Fig. S18a and detailed in Supplementary Table 3. The corresponding GRFz signal of each limb were also normalised over 200 samples and vertically concatenated to form an output matrix of size 4 × 200 used for GRFz curve prediction, as represented in Supplementary Fig. S18a. For peak GRFz prediction, the maximum value of the GRFz signals of each limb was extracted and vertically concatenated to form an output matrix of size 4 × 1 (Supplementary Fig. S18b). At the walk, the pGRFz value was defined as the second peak extracted for each limb GRFz curve^14^.

The global dataset was then randomly divided into training, validation and test subsets containing the data of 16-4-4 horses, respectively. This process avoids having the data from one horse in two different subsets, which would lead to overfitting. The strides were shuffled before training.

The minima and maxima of the training input and output signals were used to normalise the data from each dataset. In total, 960 s of walk, resulting in 672 strides were used for the walk dataset and a total of 1920s of trot, resulting in 2497 strides were used for the trot dataset (Supplementary Table 4).

### LSTM-RNN architectures and training

Prior to this work, a grid search^56^ was used to tune the hyperparameters (number of hidden units, initial learning rate, batch size) of different LSTM-RNN topologies (unidirectional and bidirectional LSTM-RNN). The bilayer bidirectional LSTM-RNN with 200 hidden-units each proved to be the most capable to predict correct GRFz curves and pGRFz values and was thus selected. The final neural networks used are presented in Supplementary Fig. S15.

Between the two bidirectional LSTM-RNN layers, a dropout layer of probability 0.40 was used to reduce overfitting risks. The second bidirectional LSTM-RNN layer was followed by a fully connected layer and a regression layer. The networks were trained with an initial learning rate of 0.002, with a batch size of 64 and the training epochs were limited to 40, to reduce overfitting.

For each gait, training and validation were repeated with 10 different input datasets, thus leading to 10 testing and evaluations of the models. For each gait and parameters, the mean and standard deviation of these testing evaluations are presented, unless stated otherwise.

### Symmetry indices

The Robinson symmetry index (SI) of the extracted and predicted pGRFz values were calculated with the Eq. (1) in this manuscript, proposed by Robinson et al.^57^.

1 $$\mathrm{SI}= 2\times \frac{{\mathrm{pGRFz}}_{\mathrm{left}} - {\mathrm{pGRFz}}_{\mathrm{right}}}{{\mathrm{pGRFz}}_{\mathrm{left}} + {\mathrm{pGRFz}}_{\mathrm{right}}}\times 100.$$

An SI of 0 shows perfect symmetry, while negative and positive SIs show a higher right and left pGRFz, respectively.

### Bland–Altman analysis

To evaluate the parameters obtained with LSTM-RNN, Bland–Altman (BA) analyses were conducted^58^. For each GRFz parameters studied (pGRFz, t-pGRFz, SI-front and SI-hind), the accuracy (bias) was defined by the mean differences between the reference value (TiF data) and the LSTM-RNN obtained parameters. The precision was defined by the standard deviation of the mean differences (STD). The 95% limits of agreement between the reference and LSTM-RNN obtained parameters were calculated as:

2 $$\mathrm{Upper \; bound \; limit }=\mathrm{ bias }+ 1.96\times \mathrm{STD},$$

3 $$\mathrm{Lower \; bound \; limit}=\mathrm{bias}-1.96\times \mathrm{STD}.$$

## Supplementary Information

Supplementary Information.

## Data Availability

The data that supports the findings of this study are available from the corresponding authors upon reasonable request.
