# Supplementary material for: Prediction of continuous and discrete kinetic parameters in horses from inertial measurement units data using recurrent artificial neural networks
Source: Sci Rep. 2023 Jan 13;13:740. doi: 10.1038/s41598-023-27899-4 (PMC9839734; doi:10.1038/s41598-023-27899-4)
Supplement: Supplementary file 1 — Supplementary Information. [file 41598_2023_27899_MOESM1_ESM.pdf]

Prediction of continuous and discrete kinetic parameters in horses from inertial measurement units data using recurrent artificial neural networks

J.I.M. PARMENTIER[1,*], S. BOSCH[2], B.J. VAN DER ZWAAG[2], M.A. WEISHAUPT[3], A.I. GMEL[4], P.J.M. HAVINGA[2], P.R. VAN WEEREN[1], F.M. SERRA BRAGANCA[1]

[1]Department of Clinical Sciences, Faculty of Veterinary Medicine, Utrecht University, 3584 CM Utrecht, The Netherlands

[2]Pervasive Systems Group, Department of Computer Science, University of Twente, 7522 NB Enschede, The Netherlands

[3]Equine Department, Vetsuisse Faculty, University of Zürich, Winterthurerstrasse 260, Zürich, Switzerland

[4]Agroscope - Swiss National Stud Farm, Les Longs-Prés, 1580, Avenches, Switzerland

[*] j.i.m.parmentier@uu.nl

SUPPLEMENTARY MATERIALS

pGRFz values

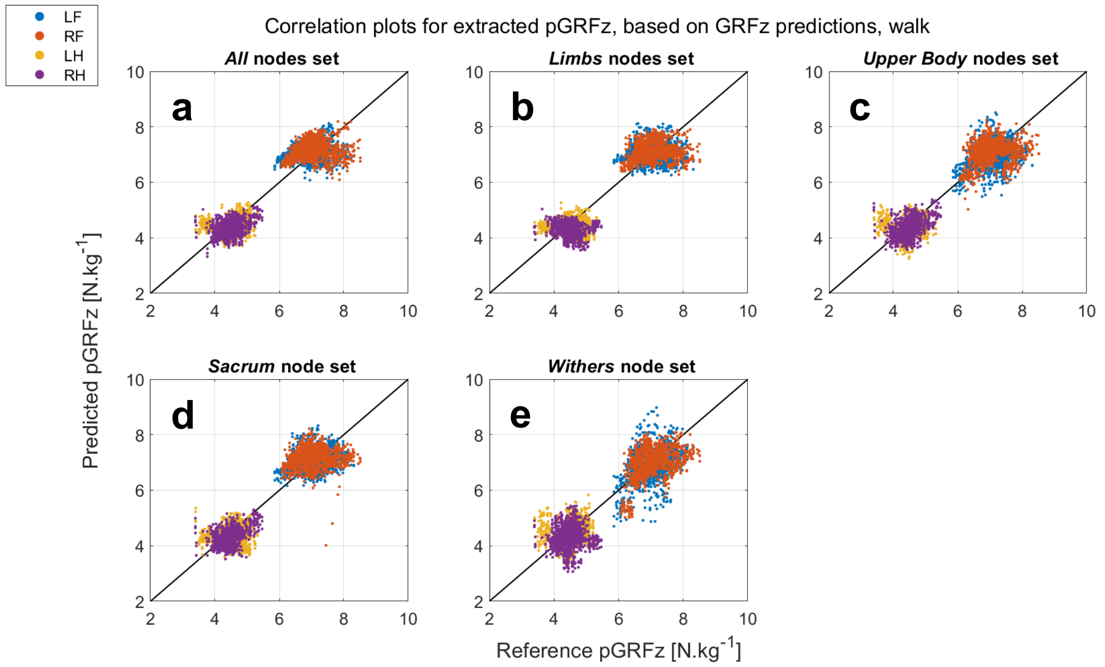

Figure S1, Correlation plots of the pGRFz extracted from the GRFz curves predicted by the different nodes sets at walk (y-axis) against the reference values extracted from the TiF data (x-axis). **a:** All (head, withers, sacrum, and limb nodes), **b:** Limbs (limb nodes), **c**: Upper-body (head, withers and sacrum nodes), **d:** Sacrum (sacrum node), **e:** Withers (withers node). LF: left front; RF: right front; LH: left hind; RH: right hind.

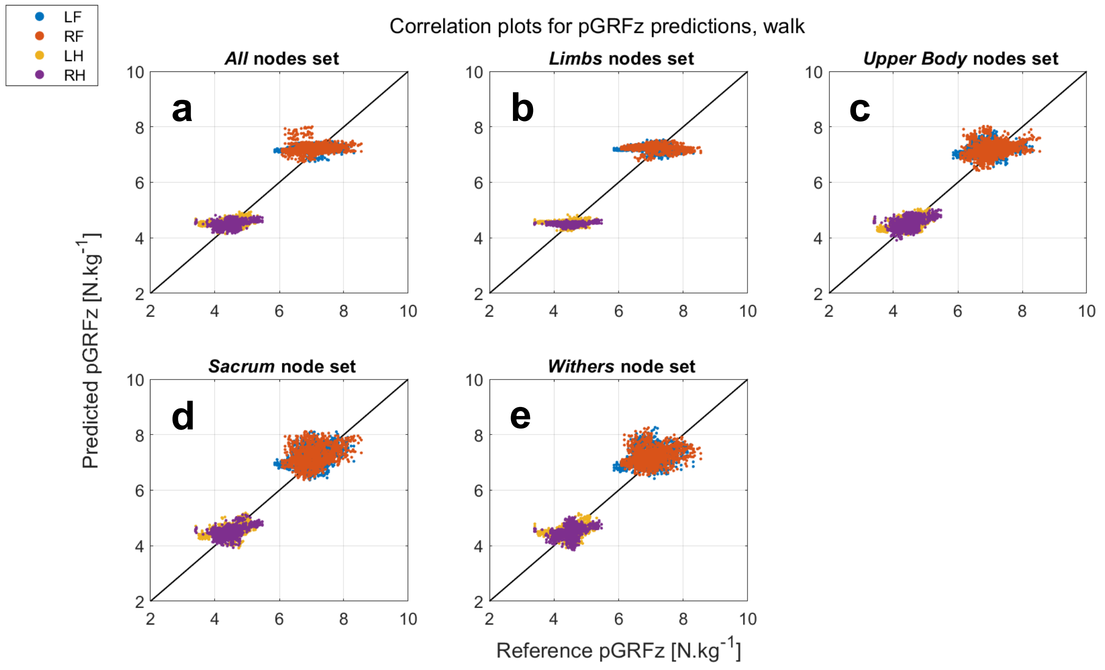

Figure S2, Correlation plots of the pGRFz predicted by the different nodes sets at walk (y-axis) against the reference values extracted from the TiF data (x-axis). **a:** All (head, withers, sacrum, and limb nodes), **b:** Limbs (limb nodes), **c**: Upper-body (head, withers and sacrum nodes), **d:** Sacrum (sacrum node), **e:** Withers (withers node). LF: left front; RF: right front; LH: left hind; RH: right hind.

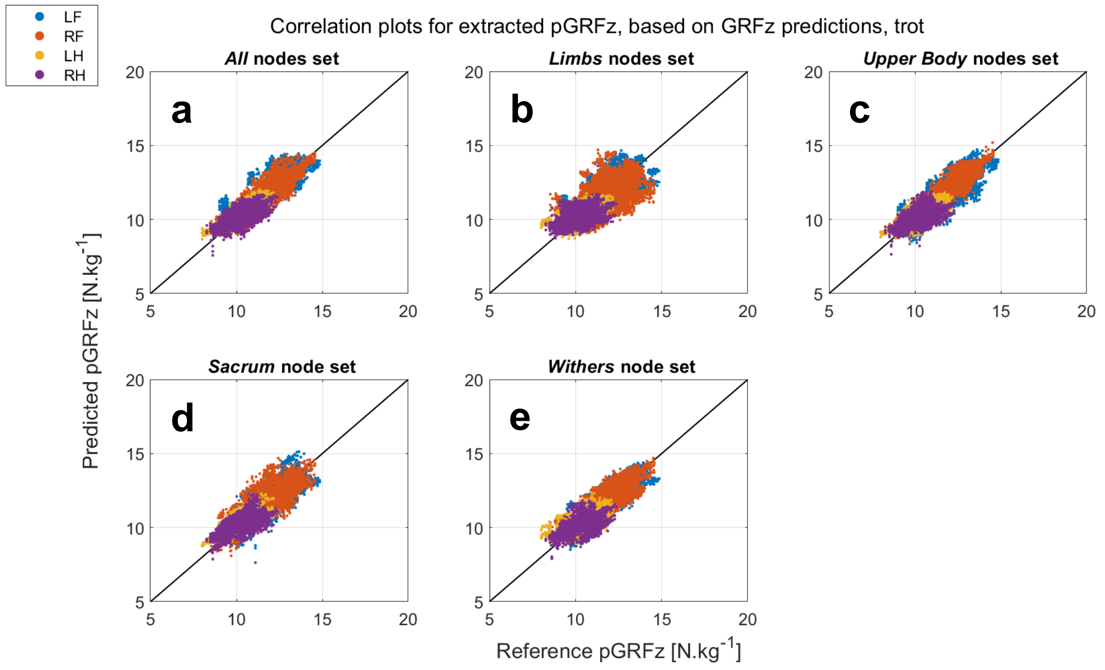

Figure S3, Correlation plots of the pGRFz predicted by the different nodes sets at trot (y-axis) against the reference values extracted from the TiF data (x-axis). **a:** All (head, withers, sacrum, and limb nodes), **b:** Limbs (limb nodes), **c**: Upper-body (head, withers and sacrum nodes), **d:** Sacrum (sacrum node), **e:** Withers (withers node). LF: left front; RF: right front; LH: left hind; RH: right hind.

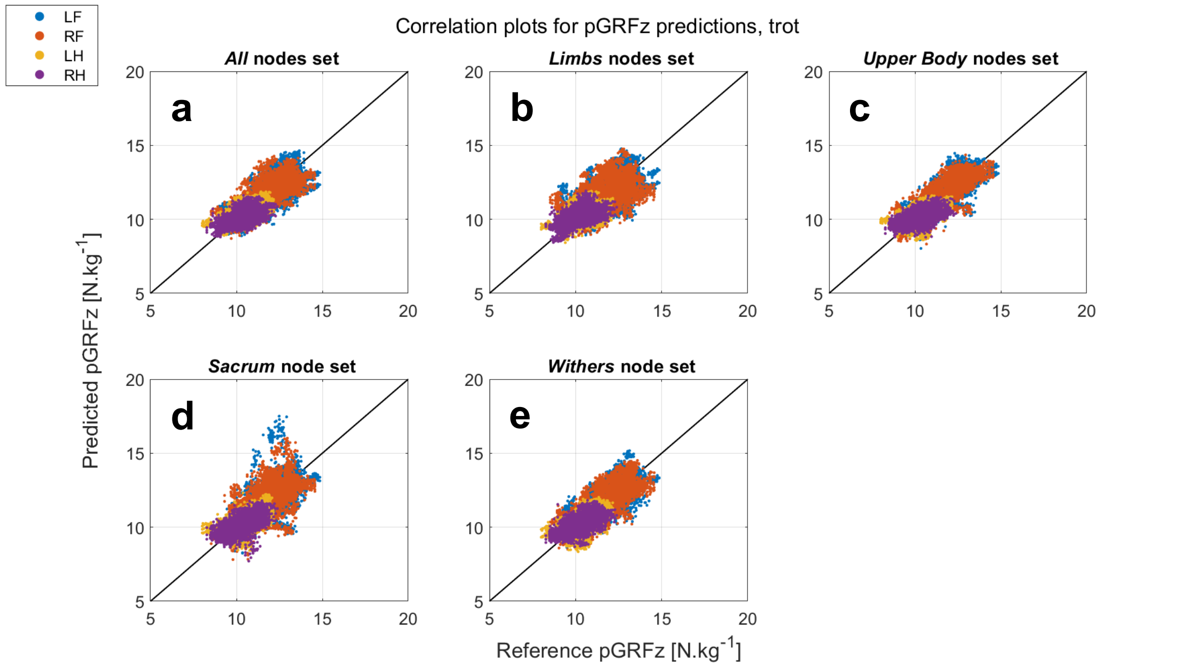

Figure S4, Correlation plots of the pGRFz extracted from the GRFz curves predicted by the different nodes sets at trot (y-axis) against the reference values extracted from the TiF data (x-axis). **a:** All (head, withers, sacrum, and limb nodes), **b:** Limbs (limb nodes), **c**: Upper-body (head, withers and sacrum nodes), **d:** Sacrum (sacrum node), **e:** Withers (withers node). LF: left front; RF: right front; LH: left hind; RH: right hind.

Time-to-peak GRFz (t-pGRFz)

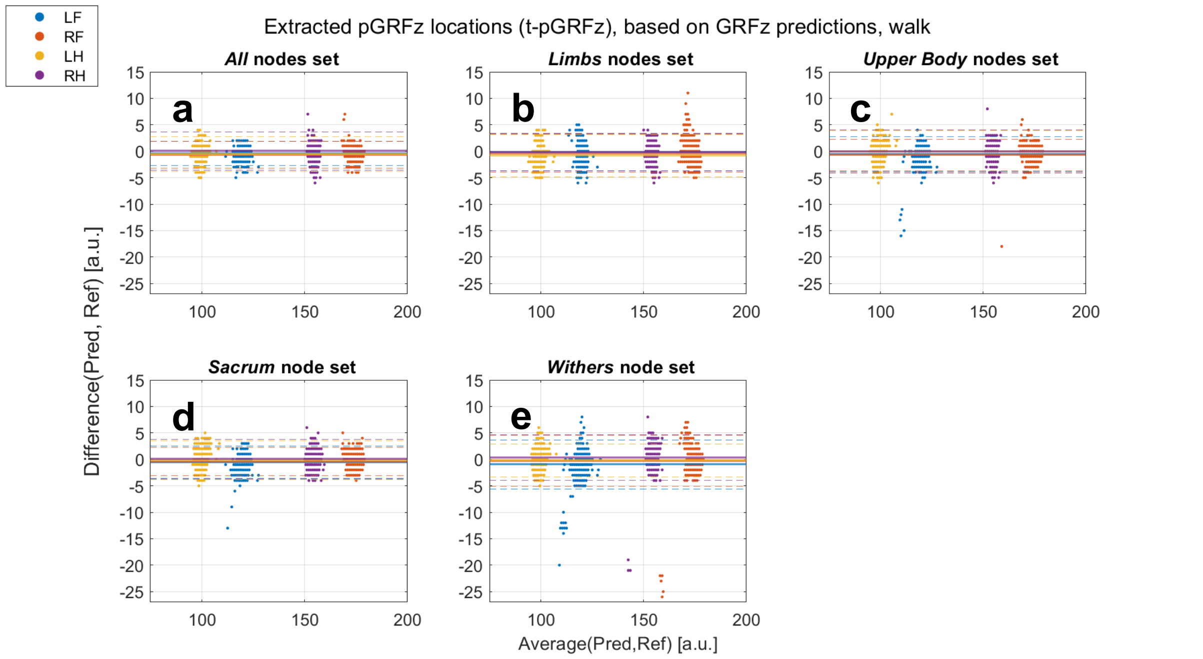

Figure S5, Bland-Altman plots of the t-pGRFz extracted from the GRFz curves predicted by the different nodes sets at walk (Pred) against the reference values extracted from the TiF data (Ref). **a:** All (head, withers, sacrum, and limb nodes), **b:** Limbs (limb nodes), **c**: Upper-body (head, withers and sacrum nodes), **d:** Sacrum (sacrum node), **e:** Withers (withers node). LF: left front; RF: right front; LH: left hind; RH: right hind.

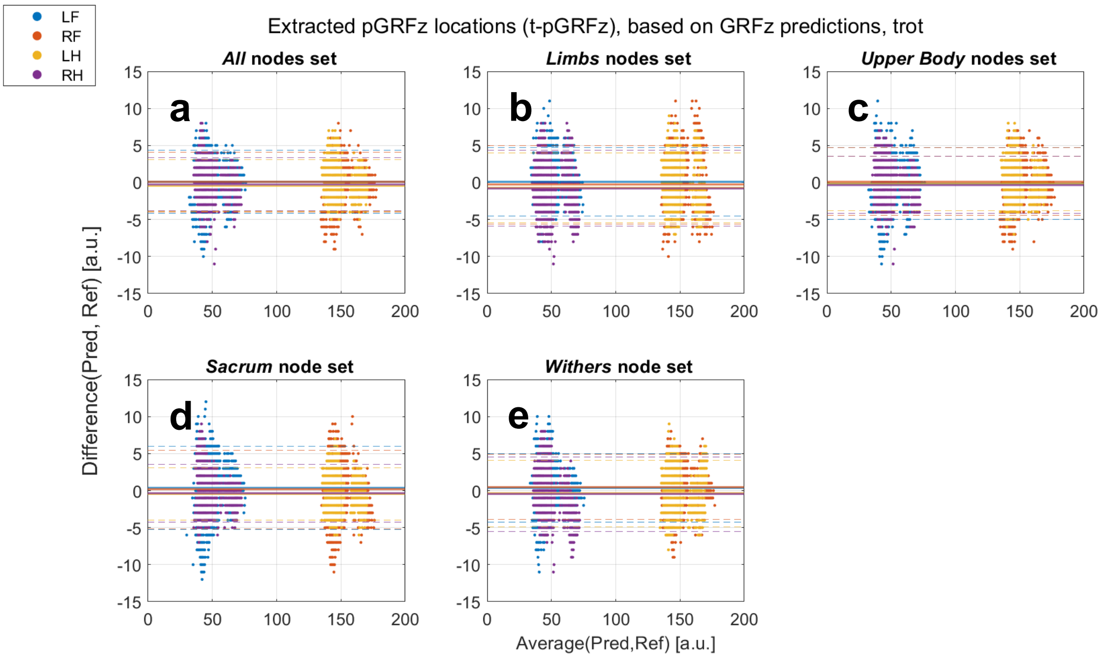

Figure S6, Bland-Altman plots of the t-pGRFz extracted from the GRFz curves predicted by the different nodes sets at walk (Pred) against the reference values extracted from the TiF data (Ref). **a:** All (head, withers, sacrum, and limb nodes), **b:** Limbs (limb nodes), **c**: Upper-body (head, withers and sacrum nodes), **d:** Sacrum (sacrum node), **e:** Withers (withers node). LF: left front; RF: right front; LH: left hind; RH: right hind.

pGRFz symmetry indices (SI)

Bland-Altman plots

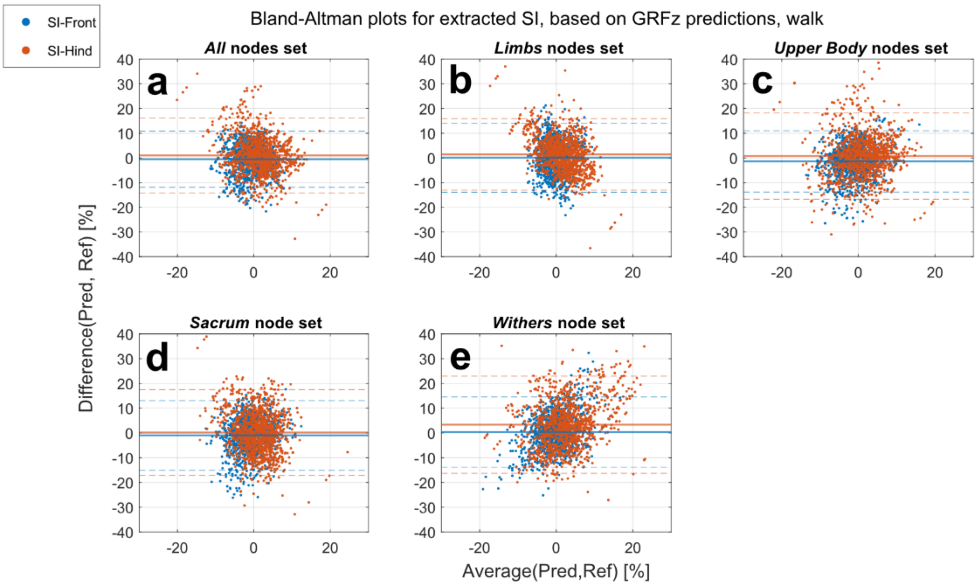

Figure S7, Bland-Altman plots of the symmetry indices (SI) based on the pGRFz extracted from the GRFz curves predicted by the different nodes sets at walk (Pred) against the reference values calculated from the TiF data (Ref): **a:** All (head, withers, sacrum, and limb nodes); **b:** Limbs (limb nodes); **c:** Upper-body (head, withers and sacrum nodes); **d:** Sacrum (sacrum node); **e:** Withers (withers node). LF: left front, RF: right front, LH: left hind, RH: right hind.

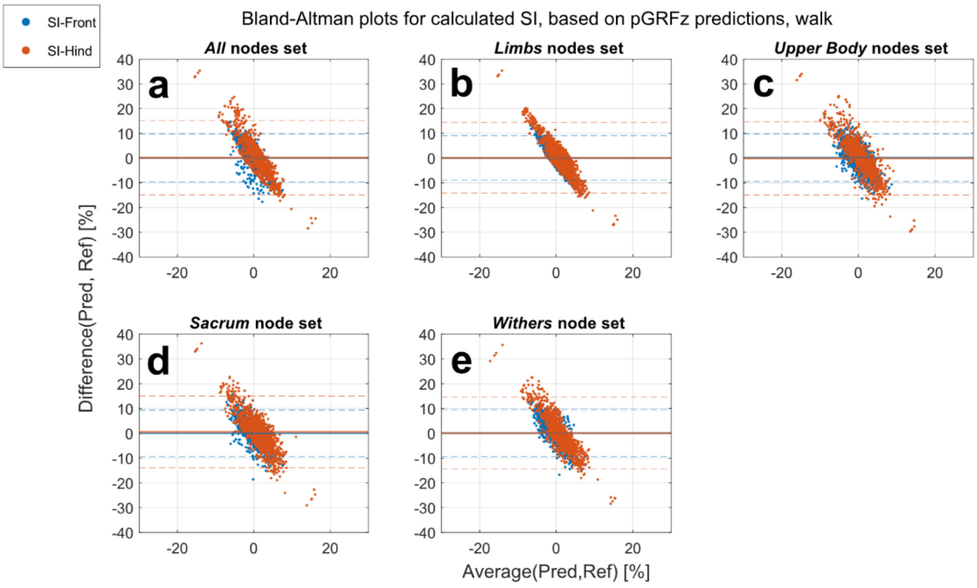

Figure S8, Bland-Altman plots of the symmetry indices (SI) based on the pGRFz predicted by different nodes sets at walk (Pred) against the reference values calculated from the TiF data (Ref): **a:** All (head, withers, sacrum, and limb nodes); **b:** Limbs (limb nodes); **c:** Upper-body (head, withers and sacrum nodes); **d:** Sacrum (sacrum node); **e:** Withers (withers node). LF: left front, RF: right front, LH: left hind, RH: right hind.

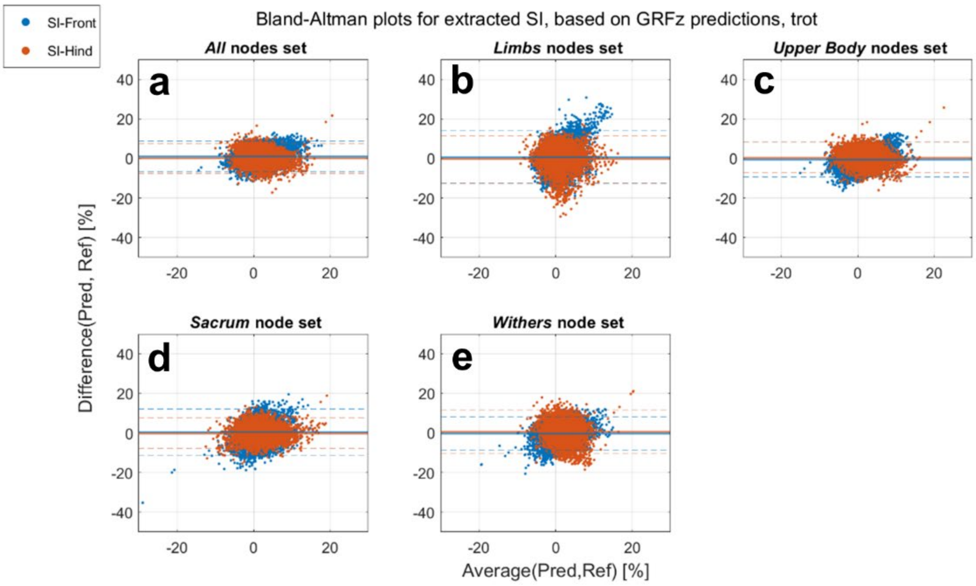

Figure S9, Bland-Altman plots of the symmetry indices (SI) based on the pGRFz extracted from the predicted GRFz curves by the different nodes sets at trot (Pred) against the reference values calculated from the TiF data (Ref): **a:** All (head, withers, sacrum, and limb nodes); **b:** Limbs (limb nodes); **c:** Upper-body (head, withers and sacrum nodes); **d:** Sacrum (sacrum node); **e:** Withers (withers node). LF: left front, RF: right front, LH: left hind, RH: right hind.

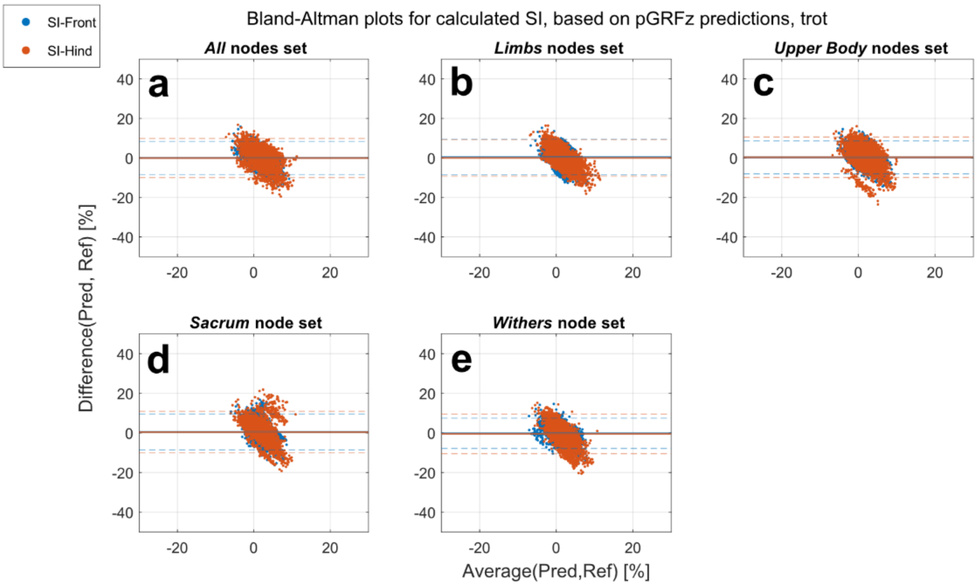

Figure S10, Bland-Altman plots of the symmetry indices (SI) based on the pGRFz predicted by different nodes sets at trot (Pred) against the reference values calculated from the TiF data (Ref): **a:** All (head, withers, sacrum, and limb nodes); **b:** Limbs (limb nodes); **c:** Upper-body (head, withers and sacrum nodes); **d:** Sacrum (sacrum node); **e:** Withers (withers node). LF: left front, RF: right front, LH: left hind, RH: right hind.

Correlation plots

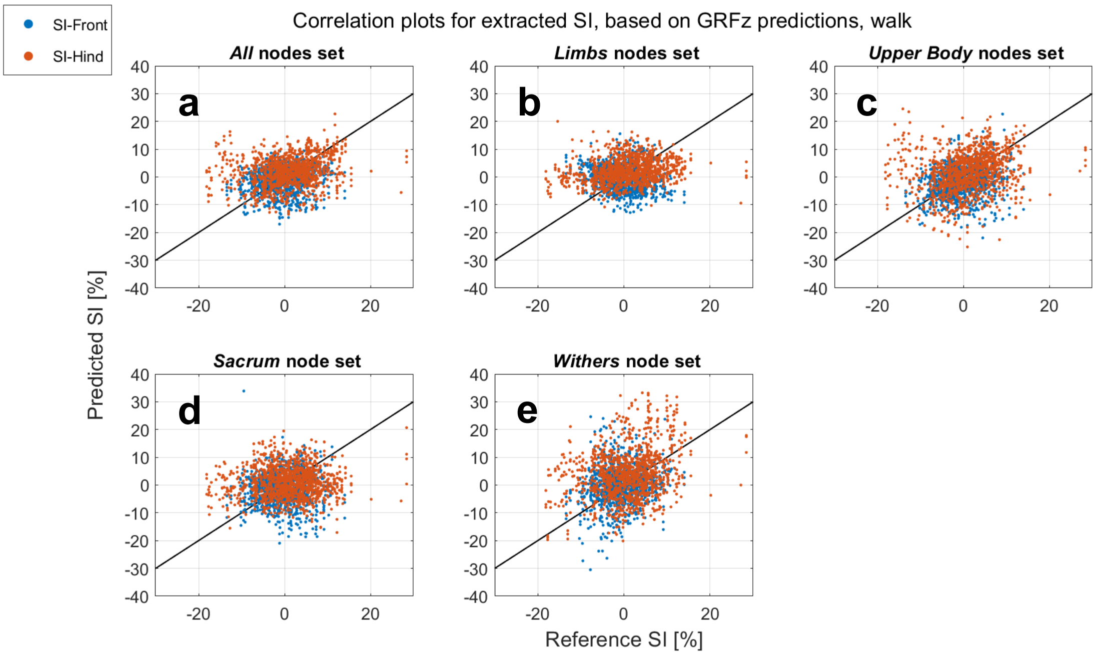

Figure S11, Correlation plots of the symmetry indices (SI) based on the pGRFz extracted from the predicted GRFz curves by the different nodes sets at walk (x-axis) against the reference values calculated from the TiF data (y-axis): **a:** All (head, withers, sacrum, and limb nodes); **b:** Limbs (limb nodes); **c**: Upper-body (head, withers and sacrum nodes); **d:** Sacrum (sacrum node); **e:** Withers (withers node). LF: left front, RF: right front, LH: left hind, RH: right hind.

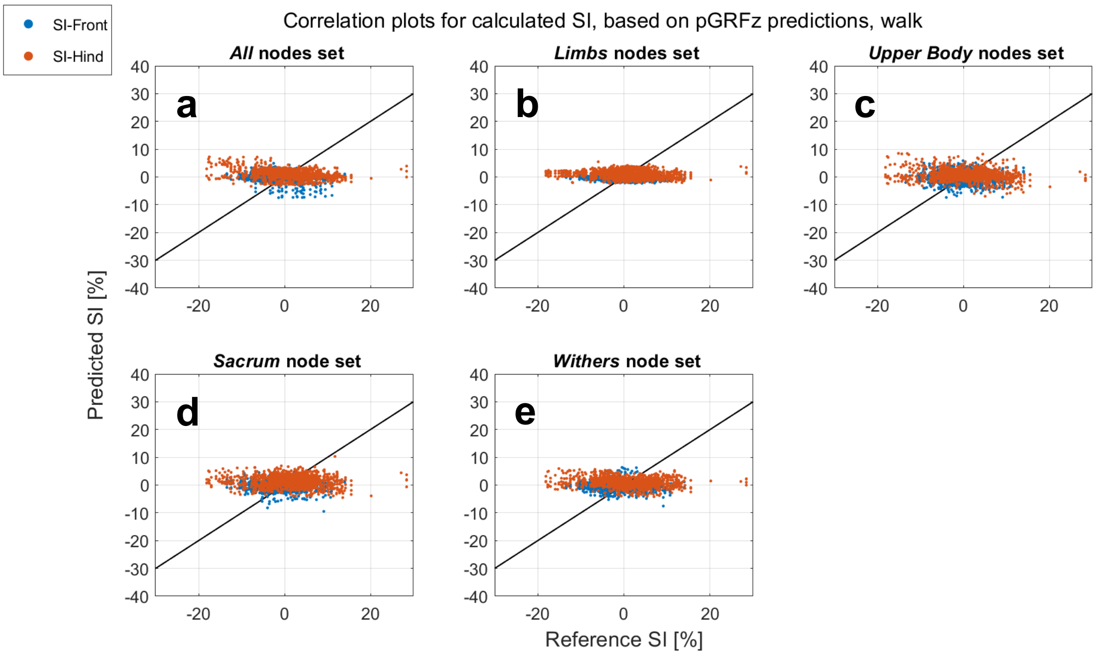

Figure S12, Correlation plots of the symmetry indices (SI) based on the pGRFz predicted by different nodes sets at walk (y-axis) against the reference values extracted from the TiF data (x-axis). **a:** All (head, withers, sacrum, and limb nodes); **b:** Limbs (limb nodes); **c**: Upper-body (head, withers and sacrum nodes); **d:** Sacrum (sacrum node); **e:** Withers (withers node). LF: left front, RF: right front, LH: left hind, RH: right hind.

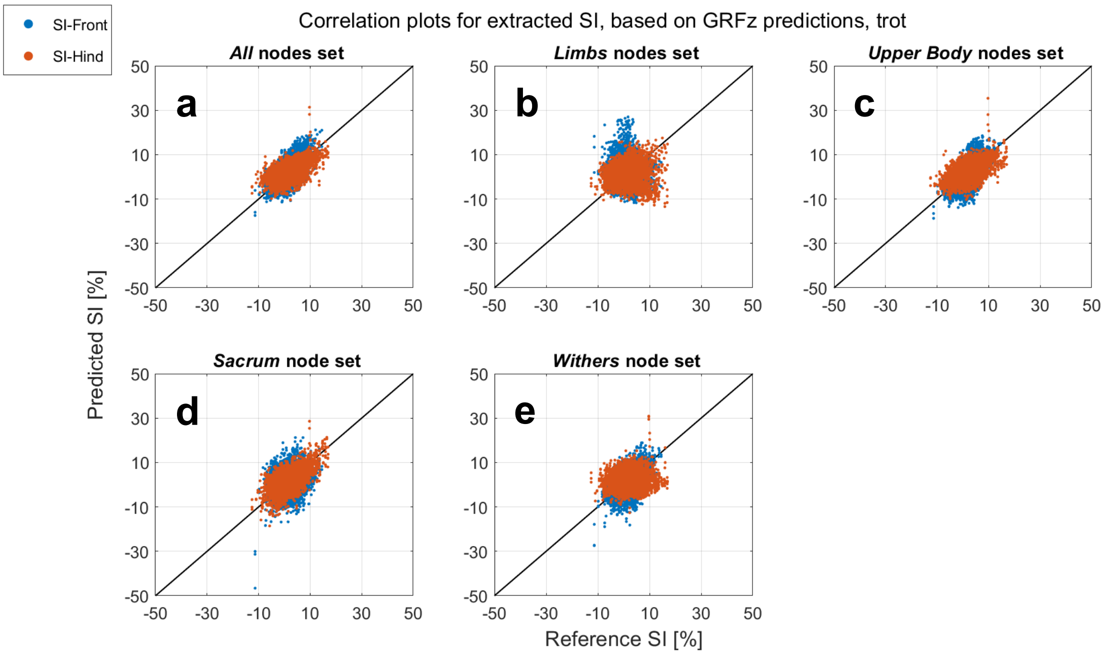

Figure S13, Correlation plots of the symmetry indices (SI) based on the pGRFz extracted from the predicted GRFz curves by the different nodes sets at trot (x-axis) against the reference values calculated from the TiF data (y-axis): **a:** All (head, withers, sacrum, and limb nodes); **b:** Limbs (limb nodes); **c**: Upper-body (head, withers and sacrum nodes); **d:** Sacrum (sacrum node); **e:** Withers (withers node). LF: left front, RF: right front, LH: left hind, RH: right hind.

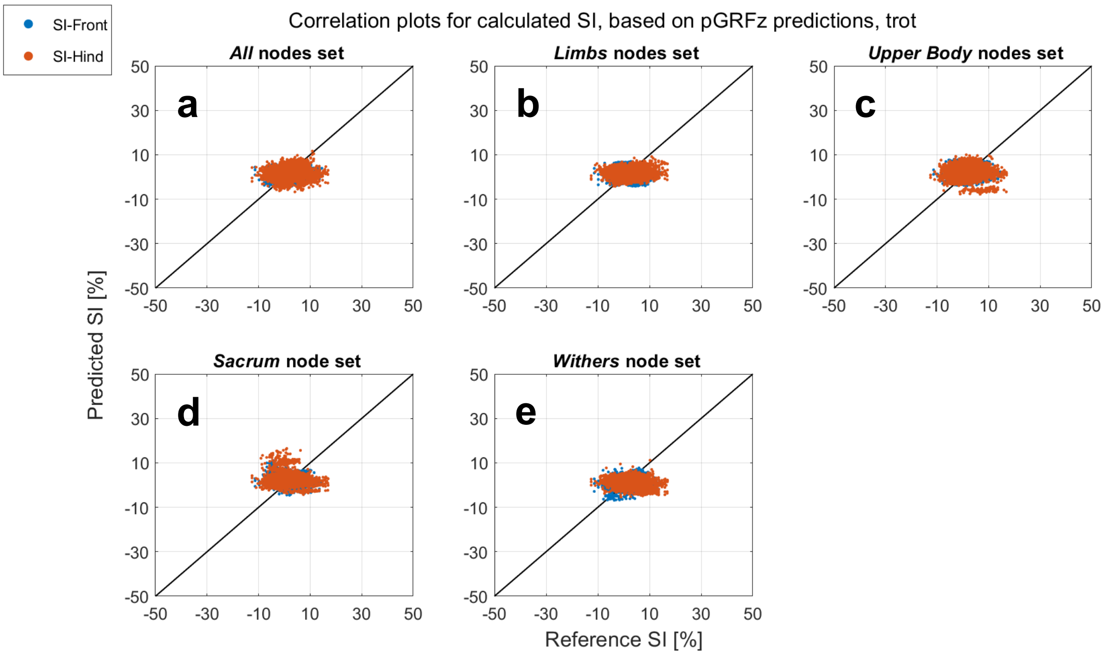

Figure S14, Correlation plots of the symmetry indices (SI) based on the pGRFz predicted by different nodes sets at trot (y-axis) against the reference values extracted from the TiF data (x-axis). **a:** All (head, withers, sacrum, and limb nodes); **b:** Limbs (limb nodes); **c**: Upper-body (head, withers and sacrum nodes); **d:** Sacrum (sacrum node); **e:** Withers (withers node). LF: left front, RF: right front, LH: left hind, RH: right hind.

GRFz curves per speed – examples

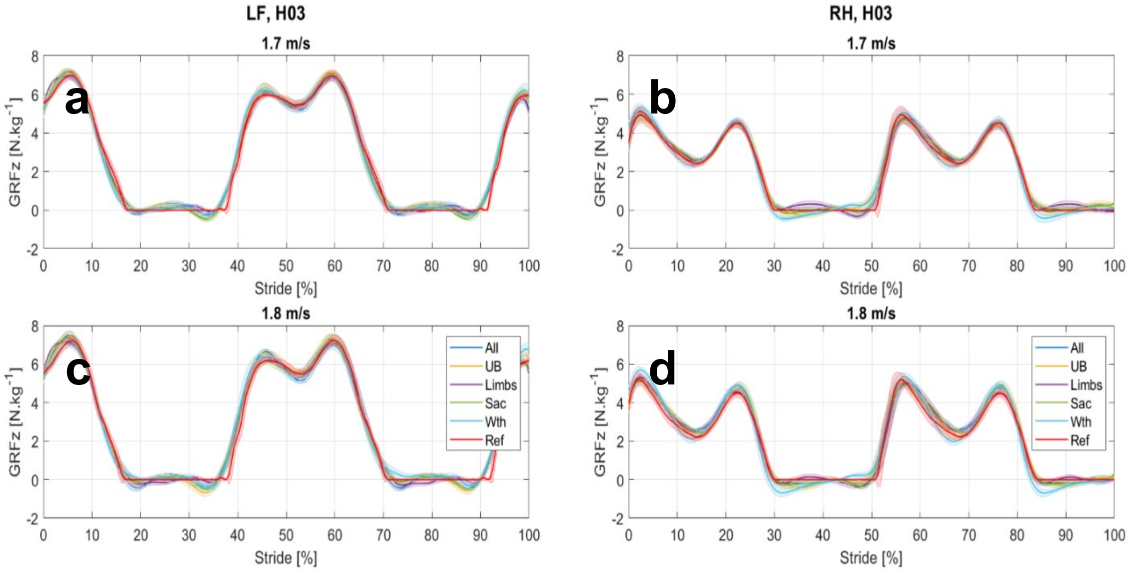

Figure S15, Example of left front (LF) and right hind (RH) GRFz curves predicted by the different nodes sets for the walk data, at all the speeds used, for one horse (H03). **a:** 1.70m/s, LF; **b:** 1.70m/s, RH; **c**: 1.80m/s, LF; **d:** 1.80m/s, RH. The average strides are displayed as a bold line and standard deviations are shown by thin lines and shaded areas. The reference curves are shown in red (Ref: TiF reference), the curves predicted by models trained with all nodes (All: head, withers, sacrum and limb nodes) in orange, with upper-body nodes (UB: head, withers and sacrum nodes) in yellow, with limb nodes (Limbs: limb nodes) in purple, with the sacrum node (Sac: sacrum node) in green and with the withers node (Wth: withers node) in light blue.

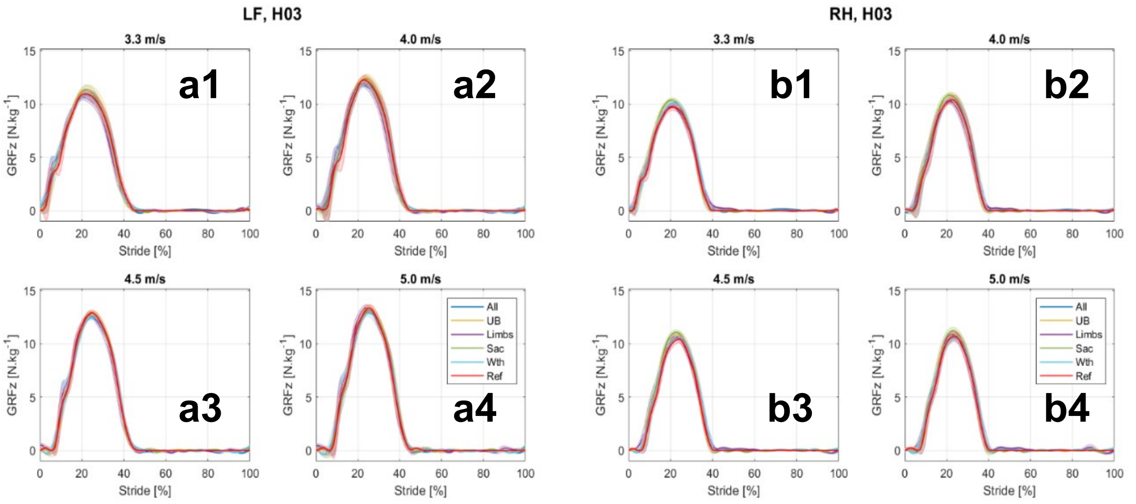

Figure S16, Example of left front (LF) and right hind (RH) GRFz curves predicted by the different nodes sets for the trot data, at all the speeds used, for one horse (H03). **a1:** 3.30m/s, LF; **a2**: 4.00m/s, LF; **a3**: 4.50m/s, LF; **a4**: 5.00m/s, LF; **b1**: 3.30m/s, RH; **b2**: 4.00m/s, RH; **b3**: 4.50m/s, RH; **b4**: 5.00m/s, RH. The average strides are displayed as a bold line and standard deviations are shown by thin lines and shaded areas. The reference curves are shown in red (Ref: TiF reference), the curves predicted by models trained with all nodes (All: head, withers, sacrum and limb nodes) in orange, with upper-body nodes (UB: head, withers and sacrum nodes) in yellow, with limb nodes (Limbs: limb nodes) in purple, with the sacrum node (Sac: sacrum node) in green and with the withers node (Wth: withers node) in light blue.

Methods illustrations

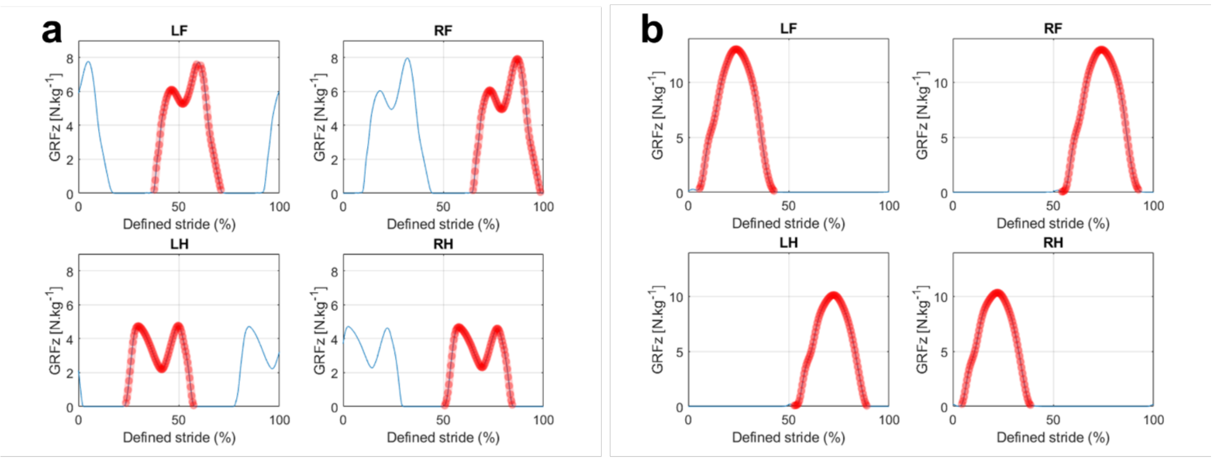

Figure S17, Window of data defined for walk (**a**) and trot (**b**), with the stance phases of interest highlighted in red.

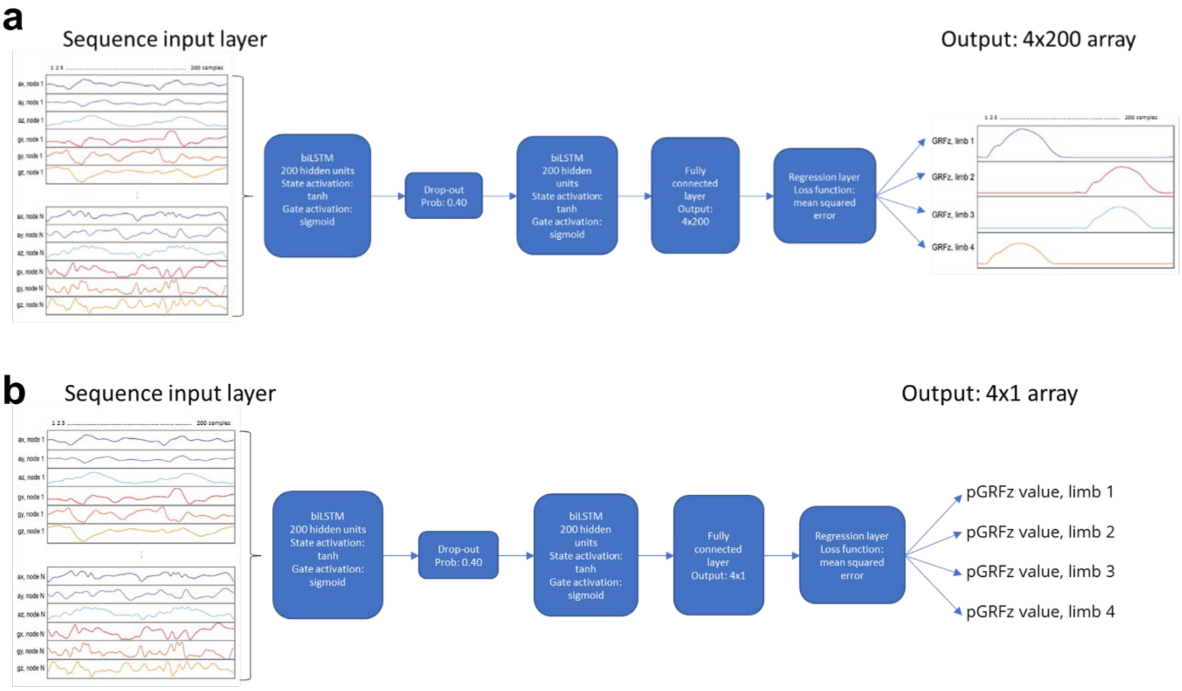

Figure S18, LSTM-RNN architectures used to predict GRFz curves (**a**) and pGRFz values (**b**). The two bidirectional LSTM-RNN layers consist of 200 hidden units and use a tanh (hyperbolic tangent) state activation and a sigmoid gate activation. They are separated by a dropout layer of probability 0.40 and are followed by a fully-connected layer of output 200x4 (**a**) or 1x4 (**b**). The regression layer uses a mean-squared-error loss function.

Tables

Table 1, Biases, confidence intervals lower (LBD) and upper (UBD) bounds and confidence intervals ranges extracted from the Bland-Altman representations of the pGRFz extracted from the predicted GRFz (Extracted), and predicted (Predicted) by the different nodes sets. The reference pGRFz values were extracted from the TiF data. All: Head, Withers, Sacrum and Limb nodes; Limbs: Limb nodes; UB (upper-body): Head, Withers and Sacrum nodes, Sac (sacrum): Sacrum node, Wth (withers): Withers node.

WALK		pGRFz Front				pGRFz Hind			
		Bias	LBD	UBD	Range	Bias	LBD	UBD	Range
Extracted	All	0,07	-0,97	1,10	2,07	-0,12	-0,84	0,59	1,43
	Limbs	0,05	-1,02	1,11	2,13	-0,17	-1,04	0,70	1,73
	UB	-0,04	-1,09	1,02	2,12	-0,12	-0,89	0,64	1,53
	Sac	0,06	-0,98	1,10	2,08	-0,17	-0,93	0,58	1,51
	Wth	-0,09	-1,21	1,02	2,23	-0,13	-1,12	0,86	1,98
Predicted	All	0,18	-0,77	1,12	1,89	0,00	-0,65	0,64	1,29
	Limbs	0,18	-0,80	1,16	1,95	0,00	-0,68	0,68	1,36
	UB	0,15	-0,82	1,13	1,95	0,01	-0,58	0,60	1,19
	Sac	0,20	-0,75	1,15	1,90	0,02	-0,59	0,63	1,22
	Wth	0,18	-0,82	1,19	2,01	0,00	-0,64	0,63	1,27

TROT		pGRFz Front				pGRFz Hind			
		Bias	LBD	UBD	Range	Bias	LBD	UBD	Range
Extracted	All	0,02	-1,21	1,26	2,47	0,04	-0,97	1,04	2,01
	Limbs	0,02	-1,87	1,92	3,79	-0,01	-1,32	1,29	2,60
	UB	0,04	-1,20	1,28	2,47	0,01	-1,08	1,10	2,18
	Sac	0,02	-1,33	1,36	2,69	0,09	-0,98	1,15	2,13
	Wth	-0,02	-1,32	1,29	2,60	-0,02	-1,23	1,18	2,41
Predicted	All	-0,03	-1,66	1,60	3,25	-0,02	-1,23	1,18	2,42
	Limbs	-0,06	-2,25	2,13	4,37	0,01	-1,29	1,30	2,59
	UB	-0,01	-1,45	1,43	2,88	-0,02	-1,26	1,22	2,48
	Sac	0,07	-1,94	2,08	4,02	-0,02	-1,19	1,15	2,33
	Wth	0,04	-1,62	1,70	3,32	0,00	-1,20	1,20	2,40

Table 2, Biases, confidence intervals lower (LBD) and upper (UBD) bounds and confidence intervals ranges extracted from the Bland-Altman representations of the symmetry indices (SI) based on pGRFz extracted from the predicted GRFz (Extracted), and predicted by the different nodes sets (Predicted). The reference SI values were calculated based on the pGRFz extracted from the TiF data. All: Head, Withers, Sacrum and Limb nodes; Limbs: Limb nodes; UB (upper-body): Head, Withers and Sacrum nodes, Sac (sacrum): Sacrum node, Wth (withers): Withers node.

WALK		SI-Front				SI-Hind				TROT		SI-Front				SI-Hind			
		Bias	LBD	UBD	Range	Bias	LBD	UBD	Range			Bias	LBD	UBD	Range	Bias	LBD	UBD	Range
Extracted	All	-0,54	-11,90	10,81	22,72	1,04	-14,15	16,22	30,38	Extracted	All	1,03	-6,61	8,67	15,29	0,07	-7,52	7,66	15,18
	Limbs	0,04	-13,94	14,02	27,96	1,40	-13,08	15,87	28,95		Limbs	0,75	-12,83	14,32	27,15	-0,39	-12,31	11,53	23,84
	UB	-1,44	-13,86	10,97	24,82	0,73	-16,75	18,22	34,97		UB	-0,54	-9,23	8,15	17,39	0,44	-7,28	8,15	15,43
	Sac	-1,02	-15,09	13,06	28,15	0,17	-17,16	17,51	34,67		Sac	0,25	-11,57	12,07	23,64	-0,15	-7,75	7,44	15,19
	Wth	0,33	-13,87	14,53	28,40	3,32	-16,29	22,94	39,23		Wth	-0,32	-8,85	8,21	17,06	0,64	-10,32	11,59	21,91
Predicted	All	0,05	-9,69	9,78	19,46	0,15	-14,92	15,23	30,15	Predicted	All	-0,01	-8,38	8,37	16,75	-0,09	-9,98	9,80	19,78
	Limbs	0,03	-8,99	9,05	18,04	0,06	-14,19	14,32	28,51		Limbs	0,41	-8,62	9,44	18,06	0,02	-9,21	9,26	18,47
	UB	0,17	-9,45	9,78	19,23	-0,14	-14,99	14,70	29,68		UB	0,29	-8,04	8,62	16,66	0,33	-9,94	10,59	20,53
	Sac	0,01	-9,35	9,36	18,71	0,53	-13,90	14,96	28,87		Sac	0,51	-8,60	9,61	18,21	0,45	-10,01	10,92	20,93
	Wth	0,09	-9,31	9,50	18,81	0,05	-14,45	14,55	29,00		Wth	-0,21	-7,87	7,46	15,33	-0,45	-10,51	9,60	20,11

Table 3, Overview of the nodes sets and signals used as input for training the LSTM-RNN networks. LF: left front limb, RF: right front limb, LH: left hind limb; RH: right hind limb

Nodes set name	Nodes used	Signals used	Total input features
All	Head, Withers, Sacrum, LF, RF, LH, RH	3D merged acceleration<br>3D rotation rate	42
Upper-body (UB)	Head, Withers, Sacrum	3D merged acceleration<br>3D rotation rate	18
Limbs	LF, RF, LH, RH	3D merged acceleration<br>3D rotation rate	24
Withers (Wth)	Withers	3D merged acceleration<br>3D rotation rate	6
Sacrum (Sac)	Sacrum	3D merged acceleration<br>3D rotation rate	6

Table 4, Data distribution per gait and speed, in duration (seconds (s)) and number of windows

	Walk		Trot			
Total data duration (s)	960		1920			
Total data (windows)	672		2497			
Speed (m/s)	1.7	1.8	3.3	4.0	4.5	5.0
Amount (s)	665.16	665.33	422.02	425.60	426.50	430.12
Amount (windows)	332	340	577	611	640	669
